# Adaptive refinement and prompt-guided conditioning for clinically realistic LLM-generated synthetic pediatric data

**DOI:** 10.3389/fdgth.2026.1826526

**Published:** 2026-08-19

**Authors:** Ebtesam Alomari

**Affiliations:** Faculty of Computing and Information, Al-Baha University, Al-Baha, Saudi Arabia

**Keywords:** large language models, synthetic data generation, electronic health records (EHRs), pediatric intensive care, length of stay prediction

## Abstract

**Background:**

The development of reliable healthcare systems requires high-quality and representative datasets. Current issues, such as limited data access, bias, and the lack of diversity in Electronic Health Record (EHR) data, limit the model's generalizability, reliability, and robustness. Specifically, generating synthetic pediatric intensive care unit (PICU) data is challenging due to high age-related variability. Subsequently, the prediction task in this domain poses additional challenges due to complex feature dependencies. Most current approaches, such as GAN-based models, do not distinguish between features and the outcome variable, so they are not designed to improve prediction. Leveraging the power of LLMs for medical data generation required careful consideration of the prompt design to preserve the underlying data distribution. Besides, it is essential to consider dependency-aware guides for the models to generate high-quality synthetic data that not only mitigates existing dataset limitations but also improves prediction performance.

**Methods:**

This work proposes an LLM-based synthetic data generation pipeline integrating a rule-guided prompting method and advanced post-processing strategies. We validate the proposed framework on the task of predicting pediatric intensive care length of stay. We introduce a structure-prompt design that includes per-class guides for the most influential features, integrating key feature–feature interactions and highly correlated feature pairs. Subsequently, the rules were formulated based on the extracted statistics and frequency distributions derived from realistic clinical data. We introduce a density-aware refinement method to ensure diversity, improve fidelity, and preserve utility. Furthermore, we employed two LLMs, which are ChatGPT and Claude, and conducted a comprehensive evaluation.

**Result:**

Both the Claude and Fine-tuned ChatGPT models demonstrated results comparable to baselines (CTGAN and TVAE) in terms of Macro-F1, PR-AUC, and MCC. Despite the reduction in sample size caused by the density-based filtering stage, the models achieved slight improvements over the raw synthetic data in utility evaluation. This indicates that the proposed post-processing strategy improved sample quality without compromising downstream performance.

**Conclusions:**

The findings demonstrate the effectiveness of integrating prompt-guided statistical modeling with density-aware adaptive refinement in addressing the data scarcity and generating realistic synthetic data in such risk-sensitive domains. Additionally, it highlights the success in supporting robust predictive modeling in data-constrained settings.

## 1 Introduction

High-quality data can be a valuable source of information. Synthetic data is artificially generated. It serves as an effective approach for addressing data scarcity and privacy constraints and advancing the development and validation of robust machine learning models (1). While promising, synthetic data must be generated carefully to preserve realism and reliability. Nowadays, the interest in Large Language Models (LLMs) has increased due to their remarkable success across natural language and data generation tasks. LLMs have emerged as a powerful technique for synthetic data generation. It helps minimize human effort in collecting, cleaning, and labeling data. Unlike traditional methods that require extensive pre-processing and explicit distribution modeling, LLMs can implicitly capture complex dependencies and leverage what they learned during pre-training, leading to context-aware synthetic data generation (2). Despite their flexibility, the quality of synthetic data deeply relies on prompt quality (3). Thus, prompt formulation is essential to ensure output quality and adhere to statistical and structural constraints, where poorly structured prompts may lead to inconsistent outputs and reduced fidelity.

The existing datasets for pediatric care are limited, imbalanced, and contain many incomplete and missing values. Furthermore, they did not reflect the complexity and diversity of the real hospital data. Synthetic data generation holds significant potential for pediatric research by mitigating privacy concerns, ethical barriers, and data scarcity. However, despite its potential, generating medical tabular synthetic data is a critical and complex task (4). The specially designed generative models, including Variational Autoencoder (VAE)-based methods such as TVAE (Tabular Variational Autoencoder) and Generative Adversarial Network (GAN)-based methods such as CTGAN, TGAN (TableGAN), and CopulaGAN, are widely used (5). These models are not efficient enough, particularly for classification tasks, where they treat all the features, including the target (outcome), equally. They do not capture the relationship between the target and the features during generation. Consequently, the GAN-based method required extensive pre-processing, which could affect the quality of the generated data.

Furthermore, generating medical tabular data is a non-trivial task due to various factors. Reflecting real-world medical scenarios, while preserving the dependency and considering high-dimensional data, is very complex. Additionally, the complex nature of vital signs and their correlation with the target make this challenging. Besides, balancing improvements in utility, fidelity, and quality of synthetic data is significant. Even though LLMs have strengths, they struggle to reflect realistic hospital data, particularly when dealing with high-dimensional features (6). This raises concerns about LLMs' capabilities. Additionally, it emphasizes the need for an approach that guides the LLM efficiently and performs additional steps to assess the quality of the generated data and discard samples that do not reflect realistic data.

One suitable application for evaluating synthetic tabular data generation methods in pediatrics is predicting length of stay (LOS). Pediatric intensive care data are both clinically important and methodologically challenging due to data scarcity, privacy constraints, complex feature relationships, and age-related heterogeneity. Accurate forecasting of pediatric patients' LOS is critical for managing hospital capacity and preventing overcrowding. Reliable LOS prediction enables improved resource allocation and more efficient planning of pediatric care services. Most LOS prediction studies are constrained to neonatal patients, leaving child and adolescent populations relatively underinvestigated and representing an important research gap (7).

This paper proposes a structured framework that leverages LLMs to generate synthetic tabular medical data. We explore its effectiveness for predicting pediatric length of stay. In this task, focusing solely on generating samples that reflect realistic feature distributions does not guarantee that they will support the prediction task. This misalignment becomes more noticeable and challenging, particularly when dealing with a critical domain-specific task with high-dimensional features and complex dependencies with the target variable. Therefore, the synthetic data generation should not be treated as an isolated generation process; it must align with the prediction task as well. To address this challenge, we consider incorporating prediction awareness into the generation process. Given the dimensionality and heterogeneity of clinical variables, we focus on the most influential features for LOS prediction rather than describing or constraining all features independently. We proposed a rule-guided prompting technique.

Prior studies have adopted conditional-generation-based methods, such as sequential conditioning (6), statistical-based conditioning (mean, Standard deviation, and range) (8). In this work, we integrated multiple rules, including correlation rules, feature–feature interaction rules, and feature importance analysis, and injected domain-specific information derived from real data. Furthermore, we improve the quality of the generated data by applying multi-step post-processing to discard unrealistic synthetic samples that are inconsistent with real patterns, thereby enhancing fidelity and utility. Additionally, we applied multiple evaluation measures to assess the quality of the generated synthetic data. To the best of our knowledge, none of the existing works have leveraged LLMs for synthetic data generation for pediatric LOS prediction.

The main contributions of this work are as follows:

- Develop a rule-guided prompting method to produce tabular medical data by incorporating a structured prompt combining key feature–feature interactions, highly correlated feature pairs, and statistical distributions.
- Propose a density-aware refinement method that dynamically adjusts histogram bins based on feature characteristics to enhance fidelity by ensuring that the synthetic samples align more closely with the density patterns of the real data.
- Perform a comprehensive evaluation of the generated synthetic data from different aspects, including fidelity, diversity, and downstream utility.

The remainder of this paper is organized as follows. Section 2 provides the literature review. Section 3 explains the methodology. In Section 4, we illustrate the results. Then, in Section 5, we provide the discussion and limitations. Finally, in Section 6, we present the main conclusions and future directions.

## 2 Related work

### 2.1 LOS prediction for pediatric

For pediatric LOS prediction, Balan et al. (9) applied a random forest algorithm to the 2016 Kids' Inpatient Database. The model achieved an accuracy of 94.15%. They identified key factors associated with longer stays, including female sex and complications at birth. Medeiros et al. (10) compared multiple machine learning-based models to predict pediatric length of stay using hospitalization data from a large Brazilian university hospital. The findings show that both random forests and support vector regressors achieve the best performance, with the random forest model minimizing the average absolute error. Kavanaugh et al. (11) examined whether neurocognitive functioning predicts pediatric LOS using chart data from 96 children. Results showed that overall neurocognition was associated with longer hospitalization.

For neonatal LOS, Yildirim and Canayaz (12) applied machine learning techniques, including logistic regression, extra trees, random forests, KNN, support vector classifier, and ensemble models to predict NICU LOS. They found that the voting ensemble method outperformed the other algorithms, achieving 96% performance. Bender et al. (13) developed prediction models using multiple factors, including birth weight, gestational age, and severity-of-illness scores such as perinatal extension (SNAPPE) and the morbidity assessment index for newborns (MAIN). The findings show that the MAIN-based model showed the best predictive performance compared to SNAPPE. Lee et al. (14) developed LOS prediction models for the neonatal intensive care unit (NICU) using data from over 23,000 neonates in US hospitals. Results showed that LOS varies strongly by birth weight, with lower birth weight associated with longer hospital stays. Paul et al. (15) explore the association between metabolic acidosis and length of stay in the NICU. Results show that acidified human milk fortifier (HMF) strongly predicts metabolic acidosis, which is associated with longer NICU stays.

For youth, Stewart et al. (16) investigated predictors of length of stay in adult mental health beds in Ontario, Canada. Results showed older age, male sex, and diagnoses such as schizophrenia, mood disorders, eating disorders, personality disorders, and intellectual disability were associated with longer hospital stays.

### 2.2 Synthetic data generation

#### 2.2.1 Synthetic data generation for pediatric

Limited research has addressed pediatric synthetic data generation using traditional methods. García-Domínguez et al. (17) evaluated GAN and WGAN models for generating synthetic pediatric diabetes data to address data scarcity. Results show that WGAN outperforms GAN. Vatche and John (18) applied a GAN-based method to generate synthetic echocardiographic data using the Pediatric Heart Network dataset. They compared synthetic data generated from imputed datasets (MICE) with data generated from datasets with missing values. Results show that GAN-generated data successfully resembles real data.

#### 2.2.2 Synthetic data generation for LOS prediction using traditional methods

A limited number of studies have explored the use of synthetic data for LOS prediction using traditional methods. Schiff et al. (19) used the MIMIC-IV dataset to generate synthetic data via CTGAN to improve hospital length of stay (LOS) prediction for rare diagnoses. They trained a classifier using the CatBoost algorithm. They found that integrating synthetic data with real data before training improves predictive performance. Other researchers (20) explored the use of GANs for generating synthetic tabular data. They evaluated whether synthetic data can serve as a proxy for real-world data in a multi-class LOS classification. Results indicate that CTGAN outperforms Copula GAN.

#### 2.2.3 Synthetic data generation in various domains using LLMs

In the medical domain, Hao et al. (21) proposed an LLMSYN pipeline that relies on Retrieval-Augmented Generation (RAG) and a Markov-based Generation Process inspired by the Chain-of-Thought (CoT) reasoning method. They employed the MIMIC-III dataset and generated EHR records. They utilized various LLMs, including GPT-3.5, LLaMA2-70B, LLaMA2-7B, Falcon-40B, Falcon-7B, Meditron-7B, BioMedLM-2.7B, and GPT Neo-2B. However, the finding indicates that the small models struggled to generate detailed synthetic EHR records due to a lack of medical domain knowledge. Other researchers (22) integrated the Medical Knowledge Graph (MKG) with LLMs (GPT-4, Llama 7b) to generate synthetic clinical notes. They focus on the ICD code prediction task, particularly codes K81 and I11. However, there is insufficient evaluation, as they rely solely on the hit@k score. The obtained hit@5 score demonstrates the potential of the generated data, while the hit@1 metrics have a zero score. Tang et al. (23) focused on improving biological Name Entity Recognition (NER) and relation extraction by employing LLMs to generate high-quality synthetic data for model training. Various widely known datasets have been used for each task, such as the Gene Associations Database (GAD) and the National Center for Biotechnology Information disease corpus (NCBI). They used ChatGPT and other fine-tuned models (BERT, RoBERTa, and BioBERT). The results demonstrate improvements in the F1-score for both tasks using the generated synthetic data. They performed a simple post-processing to filter duplicate generated entries. Similarly, Šuvalov et al. (24) generated synthetic medical data for drugs and procedures using the GPT-2 model trained on Estonian medical records. Then, they tested the capabilities of GPT-3.5-Turbo and GPT-4 in annotating these data for NER, comparing with human annotation. They found that the F1-score obtained varies with the amount of data used to train the model.

Moreover, Barr et al. (8) evaluated the ability of GPT-4o using zero-shot prompting in generating tabular clinical data. They tested two methods for generating synthetic data, that rely on qualitative descriptions of clinical parameters and on descriptive statistics (mean, standard deviation, and range) of the VitalDB dataset. To evaluate fidelity, they used only metrics that measure statistical similarity, such as two-sample t-tests, two-sample proportion tests, and 95% confidence interval (CI) overlap. So, they just capture means or proportions, not the full distribution. Kange et al. (25) utilized Llama 3.2 I with the chain-of-thought method to generate synthetic data for depression prediction. They found that a trained BERT model trained solely on synthetic data achieved lower Root Mean Squared Error (RMSE) and Mean Absolute Error (MAE) than one trained on real data. Lin et al. (6) applied multiple generation methods, including schema-based, conditional-based, and group-based approaches to generate synthetic data reflecting the eICU database using ChatGPT. They used XGBoost as a baseline model for predicting mortality levels and tested feature sets of 5–20 to examine the effect of dimensionality. The findings indicate the group-based method with all features obtains the lowest Kullback-Leibler (KL) divergence, and the highest AUC and AUPRC. Although they performed a privacy assessment on the generated data, they did not consider the privacy implications of providing real patient records directly to a commercial LLM for guidance.

Seedat et al. (26) addressed data scarcity in tabular machine learning by introducing CLLM. The proposed method integrates LLMs (GPT-4 and GPT-3.5) with a robust data curation mechanism. Firstly, the data is generated by guiding the prompt with real samples. Then, the Curator is a post-generation filtering method act as a supervised classifier to filter out the low-quality synthetic records. The conducted experiment mainly focuses on COVID-19 and demonstrates that CLLM outperforms traditional synthetic data generators. The study performs neither a formal privacy assessment nor a consideration of the implications of passing real patient records directly to a commercial LLM.

Table 1 shows a comparison of recent LLM-based synthetic data generation approaches in the medical domain. Although these studies have demonstrated promising results, several common limitations remain. Many may not consider the dependencies between features during data generation. Notably, most of them did not involve a structured post-processing strategy to improve the quality. Additionally, they mostly provide only partial evaluation across fidelity, utility, and privacy. Some studies provide part of the real patient records to a commercial LLM, which may limit deployment in privacy-sensitive healthcare scenarios. These limitations encourage the need for a comprehensive framework for generating clinically realistic synthetic tabular data. This work proposes clinically guided prompts, post-processing refinement, and comprehensive fidelity, utility, and privacy evaluation without exposing raw patient records.

**Table 1:** Comparison of LLM-based synthetic data generation studies for the medical domain based on methodological characteristics and evaluation aspects.

Ref.	Prompt strategy	Involve feature dependencies	Perform post-processing	Evaluation (F/U/P)
(21)	Markov-based Generation Process inspired by CoT	✗	✗	F, U, P
(22)	Few-shot prompting using an MKG, and real clinical note examples	✗	✗	U
(23)	Few-shot prompting using a small set of human-labeled examples	✗	✓	U
(24)	Few-shot prompting	✗	✓	U
(8)	Zero-shot with descriptive statistics means, standard deviations, ranges, and category proportions.	✗	✗	F
(25)	CoT	✗	✗	
(6)	Schema-based, conditional-based, group-based approach, and few-shot prompting using real records	✓	✗	F, U, P
(26)	Few-shot prompting using real records	✓	✓	U
This work	Statistics-guided prompting with descriptive statistics, feature correlations, clinical rules, and few-shot examples.	✓	✓	F, U, P

F, fidelity evaluation; U, utility evaluation; P, privacy evaluation.

✓ indicates that the aspect is addressed, while ✗ indicates that it is not explicitly addressed.

For domains beyond healthcare, several studies have also involved LLMs in synthetic data generation. Li et al. (27) leverage BERT and RoBERTa as synthetic data generators to evaluate their abilities across several classification tasks. They tested both zero-shot and few-shot methods employing several benchmark datasets. The results show that models trained on few-shot synthetic data outperform those trained on zero-shot synthetic data. However, the models struggle to generate classification data for high-level subjective tasks, which require deep understanding and knowledge. Kim et al. (28) introduced an approach, EPIC to investigate the use of LLMs for generating realistic synthetic tabular data. They provided a prompt-based approach, such as CSV-style formatting, to improve class balance and formatting consistency. They used six real-world public tabular datasets for various domains. The results show that EPIC achieves state-of-the-art classification performance and enhancing generation efficiency, particularly for imbalanced datasets.

Other synthetic data generation approaches have leveraged fine-tuning strategies to enhance domain-specific modeling performance. Wanger et al. (29) found that fine-tuning the Mistral-7B model with synthetic data generated by the same model improves stance detection performance. Other researchers proposed LLM-based tabular data synthesizers that rely on the pre-trained DistilGPT-2 model, such as GReaT (Generation of Realistic Tabular data) (30), TAPTAP (31), Tabula (32), and Pred-LLM (2).

#### 2.2.4 Post-processing techniques for synthetic tabular data

Dat et al. (33) filter the generated samples using a pre-trained classifier trained on real data. Lampis et al. (34) extended this method by proposing a dynamic sample filtering method called the Gap Filler pipeline (GaFi). The goal is to improve utility, particularly the Classification Accuracy Score (CAS) metric. They set a range of filtering thresholds and kept only samples with confidence at or above the threshold. Sachdeva et al. (35) focused on both utility and fidelity improvement. They applied the Comprehensive Extraction Strategy (CES) by passing synthetic data, combined with the real dataset, into the classifier. The accuracy is re-evaluated and compared with the baseline (the original data accuracy). At the end of this process, only samples that demonstrated a measurable benefit will be kept and combined with the original dataset. The second approach is the Optimal Extraction Strategy (OES). It involves training a classifier on synthetic data, and then retrieving the nearest synthetic neighbors for each test sample with a correct prediction. Other researchers (26) calculated confidence and uncertainty metrics to filter out samples that are uncertain or produce inconsistent predictions. Šuvalov et al. (24) filtered the generated synthetic texts after generation by comparing each generated text with the real training corpus using the Longest Common Subsequence (LCS). Additionally, in (36), the language model itself infers labels and filters out inaccurate or inconsistent samples. Regarding evaluation, Hernadez et al. (37) addressed the lack of standardized evaluation frameworks for synthetic tabular data in healthcare by proposing a structured assessment pipeline. The framework involves three key dimensions, which are resemblance, utility, and privacy. The validation across six healthcare datasets and four generations demonstrates the applicability of the proposed evaluation approach.

### 2.3 Research gap

In recent years, the interest in utilizing LLMs for synthetic data generation has increased rapidly. However, applications and evaluations in the medical domain remain limited, particularly for complex tabular data. Besides, the sensitivity of medical records poses an additional challenge, as sharing private patient information with LLMs to improve the quality of generated data would violate privacy. Existing studies focusing on LLMs and synthetic medical data rarely provide a deep evaluation of fidelity, downstream predictive utility, and the effects of post-processing steps. This gap limits the understanding of the LLM-based generators' ability to replicate real medical data.

Among medical data, pediatric intensive care data are highly sensitive and complex due to the heterogeneity of age groups, the diversity of developmental stages, and the density of clinical conditions. Subsequently, the data scarcity introduces additional complexity in pediatric settings. The existing public datasets for pediatrics are limited, imbalanced, have missing/incomplete values, and do not reflect the complexity of real hospital distributions.

Although applying traditional statistical and machine learning approaches to length-of-stay (LOS) prediction has been extensively studied in adult cohorts, pediatric intensive care units have not been comparatively investigated. Most prior work has focused on neonatal LOS, with limited comprehensive examination of the broader pediatric cohort. Leveraging LLMs for synthetic data generation to improve pediatric LOS prediction remains unexplored. This gap limits understanding of whether LLM-based generators can reliably follow clinical distributions, support downstream tasks, and improve pediatric LOS. This study fills this gap by conducting a multi-model comparison and evaluating synthetic data across a multi-step pipeline.

## 3 Materials and methods

**Figure 1:**
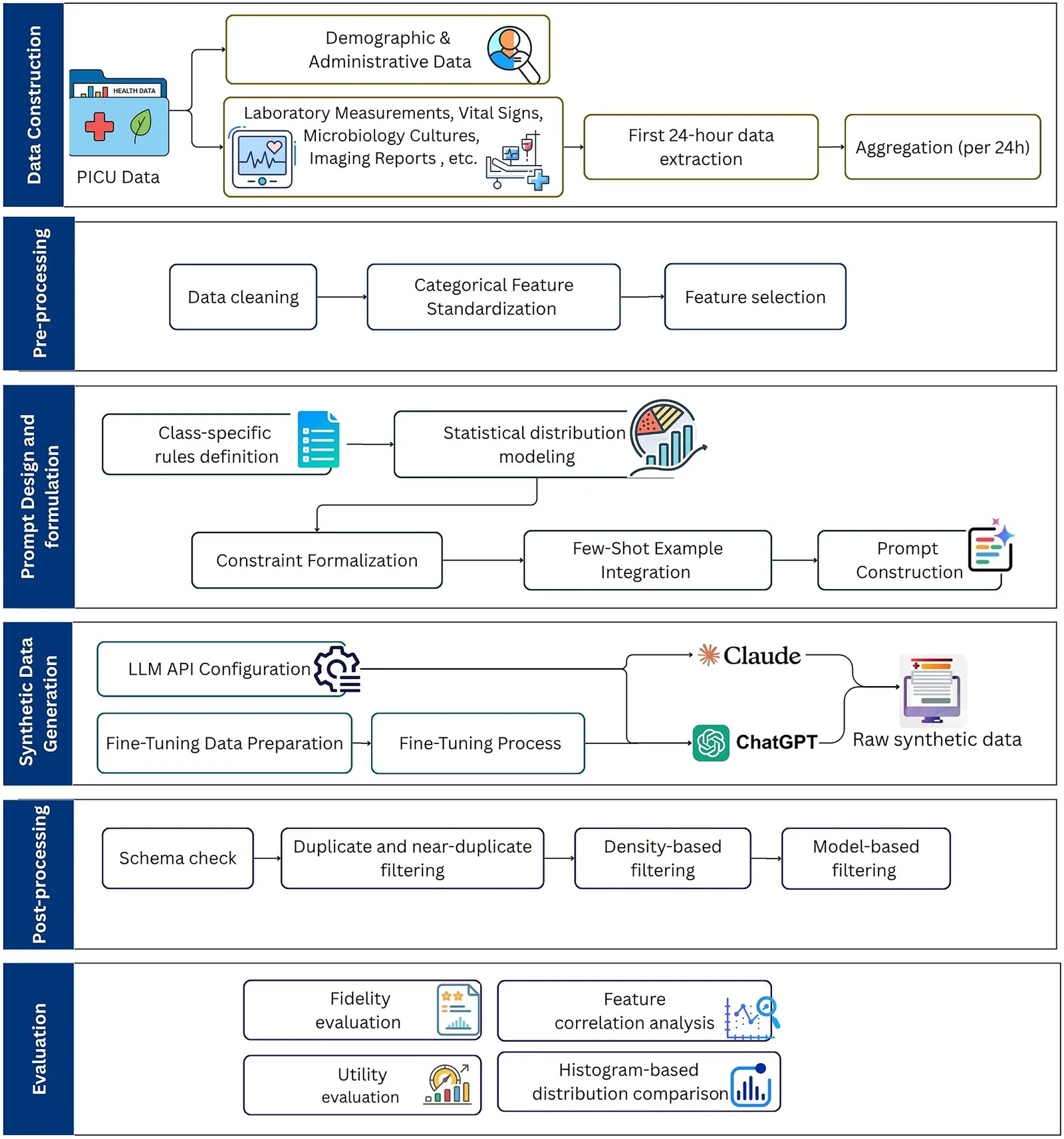
The proposed approach for synthetic data generation.

In this work, we leverage LLMs for the generation of synthetic tabular medical data. The proposed approach consists of six main components, as illustrated in Figure 1. The first component is data construction to select relevant records and aggregate data for generation. The second component is pre-processing to prepare clean and standardized data. The third component is prompt design and formulation, taking into account class-based rules and constraints. The fourth component is synthetic data generation using LLMs. The next component is applying a multi-stage post-processing pipeline to improve the alignment with real data. The last component is a comprehensive evaluation strategy that involves statistical fidelity, distributional similarity, and predictive utility. Unlike prior works that rely solely on raw synthetic data, our approach integrates density-based and model-aware refinement steps to improve quality while preserving clinically meaningful patterns. In addition, we specifically focus on improving pediatric length-of-stay prediction. However, the overall pipeline is designed to be applicable across multiple generations of synthetic data using LLMs. Furthermore, we employ two commercial LLMs. Additionally, we include TVAE and CTGAN (38) as a baseline for comparative evaluation. CTGAN is a GAN-based model, while TVAE is a Variational Autoencoder (VAE)-based method. We chose them because they are specifically designed for tabular data and widely used for generating medical data (20, 37).

### 3.1 Dataset

In this work, we utilize a Pediatric Intensive Care Unit (PICU) dataset obtained from the PhysioNet repository (39). It involves electronic health records of pediatric patients admitted to the intensive care unit (ICU). The data were extracted from the information systems of the Children's Hospital, Zhejiang University School of Medicine. They extend the approach of the widely used MIMIC (Medical Information Mart for Intensive Care) database. The dataset comprises detailed clinical records including demographic information, continuous vital signs, laboratory results, medication names, examinations, and diagnoses. The dataset contains both continuous and discrete variables, reflecting the heterogeneity of realistic medical data.

#### 3.1.1 Data integration and feature aggregation

We involved multiple relational tables from the PICU dataset, including demographic information (PATIENTS), hospital admissions (ADMISSIONS), ICU stays (ICUSTAYS), laboratory measurements (LABEVENTS and D_LABITEMS), charted clinical observations (CHARTEVENTS and D_ITEMS), microbiology cultures (MICROBIOLOGYEVENTS), imaging reports (OR_EXAM_REPORTS), and diagnosis codes (DIAGNOSES_ICD and D_ICD_DIAGNOSES). We extracted multiple patient-level features and ensured that only data recorded within the first 24 h of admission/ICU stay were used to guarantee early risk prediction. Additionally, ICD codes and their corresponding ICD chapters were extracted and encoded as structured diagnostic features. Subsequently, we filtered the vital signs and laboratory measurements to retain only records for the first 24 h, and then aggregated them to store one value for each lab or vital sign test.

One of the complex features is the microbiological culture exams/imaging exams, since we have multiple examination types for some patients and no value for others. One possible approach was to keep all cultures/imaging examinations for a patient in a single column. However, this would result in a variable-length field, which raises extra complexity in feature selection or consistency of data generation using tabular synthesis methods. The other possible approach is to represent each examination type as a separate binary feature, showing whether the examination was performed. However, this would substantially increase the number of feature dimensions and sparsity, as many examination types occur rarely. Alternatively, imaging exams were aggregated based on examination type and incorporated as a feature. Besides, we included the total number of imaging procedures per patient and the total microbiological culture counts as separate features. Although this approach reduces complexity in data generation and the number of features, it may also result in the loss of clinical information.

Moreover, the target variable is patient length of stay (LOS), measured in days. It was originally recorded as a continuous variable. Later on, we converted it into categorical features: patients who stay longer than three days are labeled as 1 (long stays), and those who stay three days or fewer are labeled as 0 (short stays). The threshold was selected based on the literature (40), enabling a clinically meaningful prediction task. Besides, the selected threshold leads to a near-balanced class distribution, which minimizes potential bias that comes from highly imbalanced classification tasks. The initial dataset size consists of 13,941 records with 47 features. Before synthetic data generation, we apply feature selection and standard pre-processing steps to handle missing values, ensure feature consistency, and exclude records with missing or invalid values.

#### 3.1.2 Ethical considerations

The PICU dataset is publicly available, and access to it requires fulfilling all the requirements and completing data usage training. The dataset contains no identifiable patient information, and all patient information is anonymized in accordance with the Health Insurance Portability and Accountability Act (HIPAA) standards. Therefore, this study did not require additional institutional review board (IRB) approval. All analyses were conducted in compliance with the data use agreement. None of the PICU dataset records were directly passed to any LLM for pre-training.

### 3.2 Pre-processing

#### 3.2.1 Data cleaning

In this step, we handle missing values and out-of-range values. Some features had a high proportion of missing values. Thus, the correlation between features and the target is calculated. We drop any feature with > 60% missing regardless of correlation. Then, we discard features with high missing values and weak correlation. We use |corr| < 0.20 as a discard threshold. This covers both weak negative and weak positive. Table 2 illustrates the features with a missing percentage above 10%. This filtering process results in discarding all 10 features represented in this table.

**Table 2:** Missing-value percentages and pearson correlation coefficients with LOS outcome.

Feature	Missing %	Correlation	Correlation group
Height	80%	0.663801	Strong Positive
Sodium	80%	−0.560087	Strong Negative
Weight	72%	−0.11846	Weak Negative
Oxygen Saturation (SpO_2_)	60%	−0.310827	Strong Negative
Systolic Blood Pressure	55%	−0.27373	Weak Negative
Diastolic Blood Pressure	55%	−0.20759	Weak Negative
Procalcitonin	52%	0.092044	Near Zero
Heart Rate	46%	0.151821	Weak Positive
Temperature	37%	−0.053495	Near Zero
Respiratory Rate	37%	0.183831	Weak Positive

Furthermore, the remaining features undergo further checks for incorrect or out-of-range data. Lab/vital test values have been carefully checked to ensure the handling of mixed units. Several laboratory features were reported in mixed measurement units (e.g., Hemoglobin in g/dL vs. mmol/L and Glucose in mg/dL vs. mmol/L). Thus, we perform unit conversions to ensure consistency and clinical validity.

Additionally, we use clinical threshold ranges specific to pediatric intensive care units (PICU) from the American College of Clinical Pharmacy (ACCP) (41) to guide the conversions and identify out-of-range data. This step helps in improving the quality of lab data for downstream analysis and modeling. Besides, we found that the percentage of missing values decreased slightly as a result of dropping records with outliers, since these records also included missing values. Apparently, those records were not helpful, as some features are incorrect and others are missing. Moreover, missingness in the aggregated 24 h lab values usually indicates that no lab was performed that day. However, records with missing values were dropped to avoid issues with data generation.

#### 3.2.2 Categorical feature standardization

The categorical features have been cleaned to improve consistency and prevent the creation of artificial categories during synthetic data generation. For instance, the ADMISSION_DEPARTMENT field's values consist of numeric suffixes [e.g., “Hematology department (1)”, “Nephrology department (2)”] and combined departments using slashes (e.g., “Orthopedics/Neurology department”), which refer to a combined administrative service. The numeric suffixes have been removed to merge identical departments under a single standardized name. Further, the combined departments have been manually reviewed, and a single category has been assigned, as splitting them without additional clinical context could introduce errors. Subsequently, to avoid sparse categories, the rare departments with fewer than 30 records have been identified for merging with other departments if they are related; otherwise, they are dropped to reduce noise.

#### 3.2.3 Feature selection

We apply the Random Forest (RF) algorithm for feature selection. We selected this algorithm for its ability to capture non-linear relationships and feature interactions, as well as to provide feature importance rank. In addition, Random Forest is robust to multicollinearity and has been widely used for feature ranking in clinical prediction tasks (42).

Before passing the data to the algorithm, we drop features that are not valid predictors of LOS from early data and do not reflect patients' status in the first 24 h, such as LAST_CAREUNIT, which indicates where the patient ended up. The dataset includes identifier variables (e.g., “ICUSTAY_ID”). All identifiers were removed to preserve patient privacy and prevent the disclosure of sensitive information. Additionally, we exclude features related to time or used to calculate the LOS, including “INTIME”, “OUTTIME” and “DOB”. Then, we apply the Random Forest selector to the remaining 27 features. A Random Forest classifier was first optimized using RandomizedSearchCV. The goal is to test different combinations of the number of trees, maximum tree depth, minimum leaf size, and feature sampling strategy. Subsequently, the best-performing model was selected to estimate feature importance scores. The optimal RF configuration consisted of 200 trees and a minimum leaf size of 2. The median importance score, which is 0.033, was used as the selection threshold. So, only features with importance values greater than or equal to this threshold were included in the further analysis. This process reduced the feature set from 27 to 10 variables. Additionally, the final dataset after pre-processing steps consists of 9,220 records. The selected features by RF are illustrated in Table 3.

**Table 3:** The selected clinical features.

Feature	Data type	Description
Age	Numerical, continuous	Patient age at admission
Micro Cultures Count	Numerical, discrete	Total number of microbiological culture tests performed within the first 24 h
Imaging Total Count	Numerical, discrete	Total number of imaging examinations conducted within the first 24 h
Albumin	Numerical, continuous	Serum albumin level measured within the first 24 h
Bicarbonate	Numerical, continuous	Blood bicarbonate level measured within the first 24 h
Chloride	Numerical, continuous	Serum chloride level measured within the first 24 h
Glucose	Numerical, continuous	Blood glucose level measured within the first 24 h
First Care Unit	Categorical	Initial ICU care unit assigned to the patient
Admission Department	Categorical	The hospital department responsible for the patient at admission
Los Category	Categorical	Categorized length of stay group

### 3.3 Structured prompt design with rule integration

We introduce a rule-guided prompting technique that incorporates statistical distribution modeling and the definition of class-specific rules. This approach supports guiding the model towards producing synthetic samples that better align with the underlying data distribution, thereby reducing unrealistic values. Statistical distribution modeling refers to the process of extracting statistical characteristics from a real dataset and encoding them later with the constraints in the prompt. These characteristics comprise class-conditional feature ranges (minimum–maximum values), categorical frequency distributions, central tendency (mean and median), and distributional quantiles. In addition to feature properties, the framework incorporates inter-feature dependencies using correlation coefficients and joint mutual information (JMI) to capture linear and nonlinear relationships between features. After that, in the class-specific rules definition component, these properties will be translated into rule-based constraints embedded in the prompt. Rather than relying on a fixed prompt, this design enables the prompt to adapt to different datasets.

**Figure 2:**
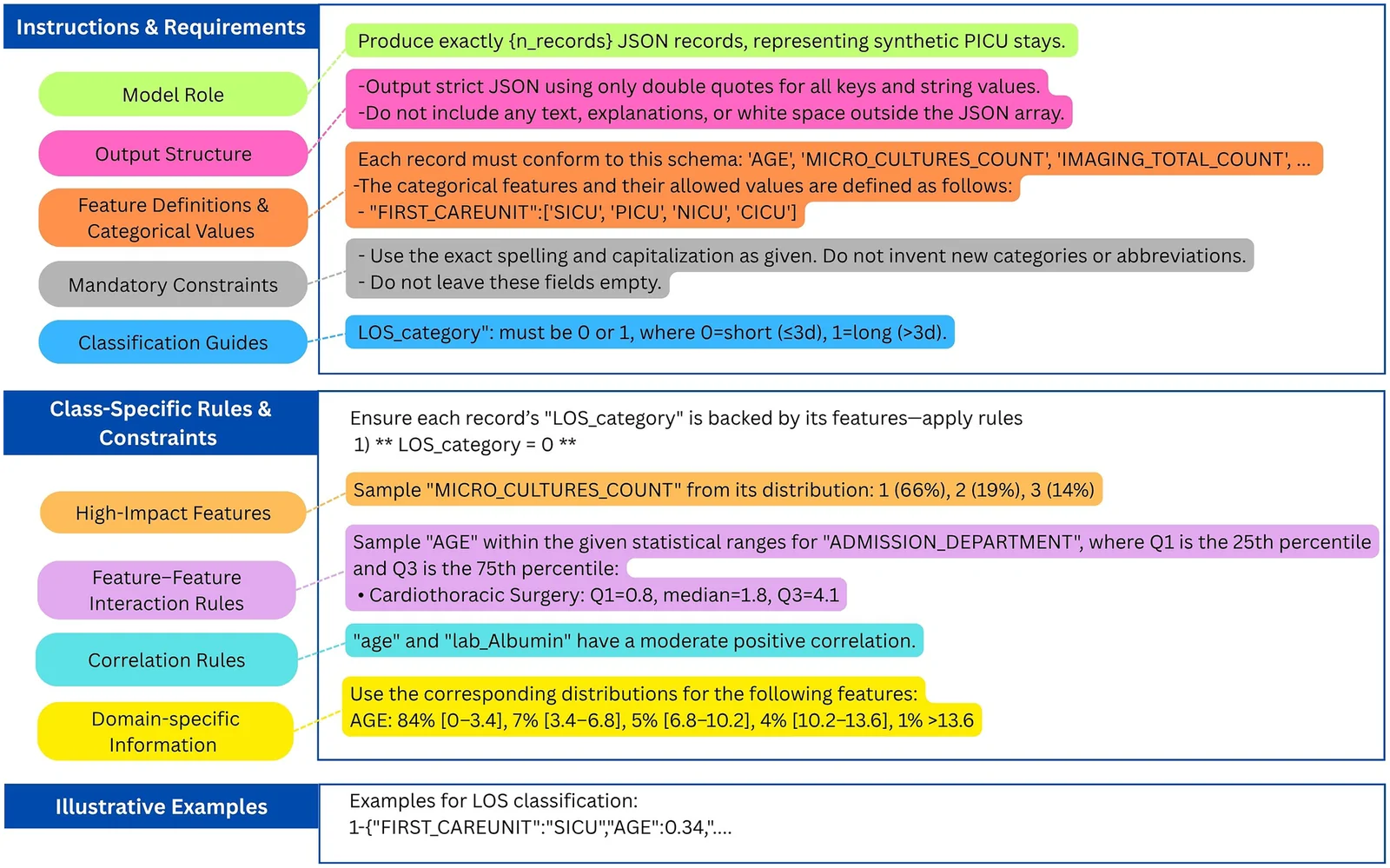
Representative example of the prompt structure with highlighted components.

The proposed prompt is decomposed into three structured functional components. Figure 2 provides a representative example of the prompt structure, highlighting its modular components. In the first component, we explicitly list the mandatory constraints and the generation requirements, including the format (JSON), the list of features, and the acceptable categories for the categorical features. Then, we provide classification guides.

The second component is the core component, which includes the class-specific constraints. Firstly, we identify the most influential features that strongly affect the prediction to facilitate more accurate data generation, rather than treating all features as equally important for prediction. Since the Random Forest algorithm was used for feature selection, we use it for importance ranking. It can involve both numeric and categorical features. Besides, it has a built-in function to assess feature importance. We list the features' importance and select the top features to highlight in the prompt later. Secondly, we check the interaction between the features. We compute the joint mutual information (JMI) to measure the amount of information that pairs of features provide, considering the target variable (43), where higher values mean the feature pair jointly provides stronger predictive information regarding LOS. It is computed as:

$$I((X_{i},X_{j});Y)$$

where $X_{i}$ and $X_{j}$ are features, *Y* is LOS.

To avoid imposing excessive constraints and introducing noise, we select the top influential pair of features that jointly influence the prediction rather than acting in isolation. We highlighted them later in the prompt as feature–feature interaction rules. For instance, we found that the combination of the child's age and the ADMISSION_DEPARTMENT capture patterns leads to different LOS predictions.

Additionally, FIRST_CAREUNIT and ADMISSION_DEPARTMENT together describe patient flow and illustrate the diversity of the case mix, which is informative for LOS prediction. For feature interactions, we reported key quantiles for the continuous numerical features and frequency distributions for discrete numerical and categorical features. Thirdly, we provide the significant pair correlations to capture key dependency patterns in the data. Fourthly, we integrate domain-specific information for each selected feature per class. The statistical information is obtained from the real data.

In the last part, we incorporate a few-shot dataset to guide the model in generating JSON format and, most importantly, in classification. To preserve privacy and prevent the leakage of any real data, we use data generated by the CTGAN. We pick the top sample that closely represents each class. Accordingly, we use NearestNeighbors and pick the top samples per class with the smallest nearest-neighbor distances. To preserve privacy, before selecting samples, we ensure that none of the GAN-generated records match real records. Subsequently, we perform a final manual check of the selected samples to ensure they comply with the rules specified in the prompt. We found that 3 samples per class were enough as an extra guide for the model.

### 3.4 LLM-based synthetic data generation

We selected two commercial LLMs. The first one is Claude Opus-4-1, released on 5 August 2025 by Anthropic (44). It excels at following complex, multi-step instructions, making it a strong candidate for the synthetic data generation task, besides other strengths, such as a large context window and high-quality reasoning. The second model is ChatGPT, introduced by OpenAI (45). Its demonstrated capabilities and widespread adoption, as well as the proven effectiveness of fine-tuning to adapt to a domain, make it a strong candidate for domain-specific tasks. We select GPT-5 mini, released on 7 August 2025. It is a lightweight, fast model, with heavier models beating previous GPT models on many intelligence tasks. For the fine-tuning process, we selected GPT-4.1 mini, as GPT-5 was not available for fine-tuning when the experiments were initiated.

Moreover, considering the stochastic nature of synthetic data generation, multiple independent generation runs were considered for each model to improve the reliability and consistency of the evaluation. In each run, a target of 4,000 synthetic samples was generated per model before applying the post-processing stages. Three runs were conducted for ChatGPT and Fine-tuned ChatGPT. However, due to the substantially higher generation cost of Claude Opus-4-1, only two independent runs were performed for that model. The reported results for evaluation correspond to the mean performance across these runs, with confidence intervals.

Before synthetic data generation, the LLM API was configured to ensure controlled, efficient output. This configuration phase involved specifying the model version, writing system-level instructions, defining the response formatting constraints, and setting generation parameters such as temperature and maximum token length. Temperature was carefully adjusted to balance diversity and stability. We tested various temperature values from 0.2 to 0.8. Lower values led to highly repetitive generated samples, while high values increased the risk of breaking the given rules. Moreover, output length is restricted by the maximum token capacity of the chosen model version. To address this issue and collect larger synthetic datasets, an iterative API call was implemented, where each call returns a specific number of structured records. After that, the outputs were aggregated to form the complete synthetic dataset. For Claude, the synthetic data were generated using claude-opus-4-1-20250805 through the Anthropic API, with a maximum output length of 8,000 tokens and a temperature of 0.2. Besides, for ChatGPT experiments, gpt-5-mini-2025-08-07 was used, while gpt-4.1-mini-2025-04-14 was used for Fine-tuning experiments, which was the latest OpenAI model supporting fine-tuning at the time of the experiment. Notably, GPT-5 models use the newer responses API parameters, which do not support configuring Temperature.

### 3.5 Post-processing pipeline

#### 3.5.1 Schema check, duplicate, and near-duplicate filtering

First, we apply a schema check to drop rows that violate the schema. We ensure that generated records include the same column names. Subsequently, we perform a hard validity check to filter out records that include outliers for numerical features or new categories for categorical features. Secondly, we filter out duplicate records, as the data was retrieved via multiple calls due to a token limit, resulting in some duplicates. Thirdly, to find near-duplicate synthetic records, we apply a random projection scheme, SimHash. It is a Locality-Sensitive Hashing (LSH) schema for measuring the cosine similarity ratio between two vectors (46). This method has been widely used to address the deduplication problem (47).

Given a dataset of *n* features $R=\{x_{1},x_{2},\ldots ,x_{n}\}$, each record $x_{i}\in R^{n}$ is converted into a set of tokens. Then it is mapped to a binary fingerprint using the SIMHASH64 function, which returns a 64-bit hash as an int.

$$h_{i}=\mathrm{SimHash}(x_{i})$$

The LSH method is applied to split the hashes into bands per class *c* to find neighbors. To measure the difference between two binary hashes, the Hamming distance is calculated as follows:

$$d_{H\;}(h_{i},h_{j})$$

Then, a threshold value *τ* is used to flag records with distance: $d_{H}<\;\tau$ as near-duplicates. Subsequently, the records are clustered into duplicate groups. Within each duplicate group, only one representative record is kept; the other are dropped.

#### 3.5.2 Density-aware refinement and model-based filtering

In density-aware refinement, we primarily focus on removing synthetic records that most closely deviate from the real data distribution. This process enhances the fidelity of synthetic data, thereby improving its utility. We apply the adaptive Wasserstein Distance (WD) filtering per-class method. We adopt WD, a feature-wise function, as a measure of the difference between real and synthetic data, given its suitability for assessing distributional fidelity.

We compute WD between real and synthetic samples for each feature per class. Then, we checked the mean class-wise WD value iteratively $\left(WD_{m}\right)$ with the assigned target $\left(WD_{t\;}\right)$. If it is less than the $WD_{t}$, we build histograms with *b* number of bins for real and synthetic data, one numerical feature at a time. To identify the optimal number of bins, we build a function that assigns a dynamic value based on the feature's characteristics. For features with a few identical values (fewer than 10), such as IMAGE_COUNT and CULTURE_COUNT, we assign the bin number equal to the number of unique values. For features with identical values between 10 and 20, such as AGE, we find that assigning the bin number to twice the number of unique values improves evaluation results. Besides, for a larger range of values, such as in lab tests, we test various bin selection rules, including 'sturges', “fd”, “doane”, “auto”, and 'scott'. We find that 'scott' improves utility results. The rate of dropped records will be dynamically adjusted from a predefined value. Then the rate is modified based on the gap between the new $WD_{m}$ and the $WD_{t}$. If the $WD_{m}$ is greater than the defined critical gap value, the drop rate will be high; otherwise it will be adjusted to be lower.

We build a density histogram for each feature to find whether the synthetic data overrepresents or underrepresents the real data. To do so, we find the identical bin edges for feature f. Then, we calculate the ratio of the density $\left(R_{\;f,E}\right)$ per-edge by dividing the synthetic density by the real density. A density deviation score φ will be assigned to all the synthetic records if the ratio is higher than the real. Then, the average of the scores across all the features will be assigned to each record. The synthetic sample with the highest φ will be discarded based on the drop rate percentage. This filtering process is iterative until the target WD is reached; thus, the remaining records are passed to the utility test. Algorithm 1 illustrates the steps.

Algorithm 1 **Proposed Density-aware Refinement.**

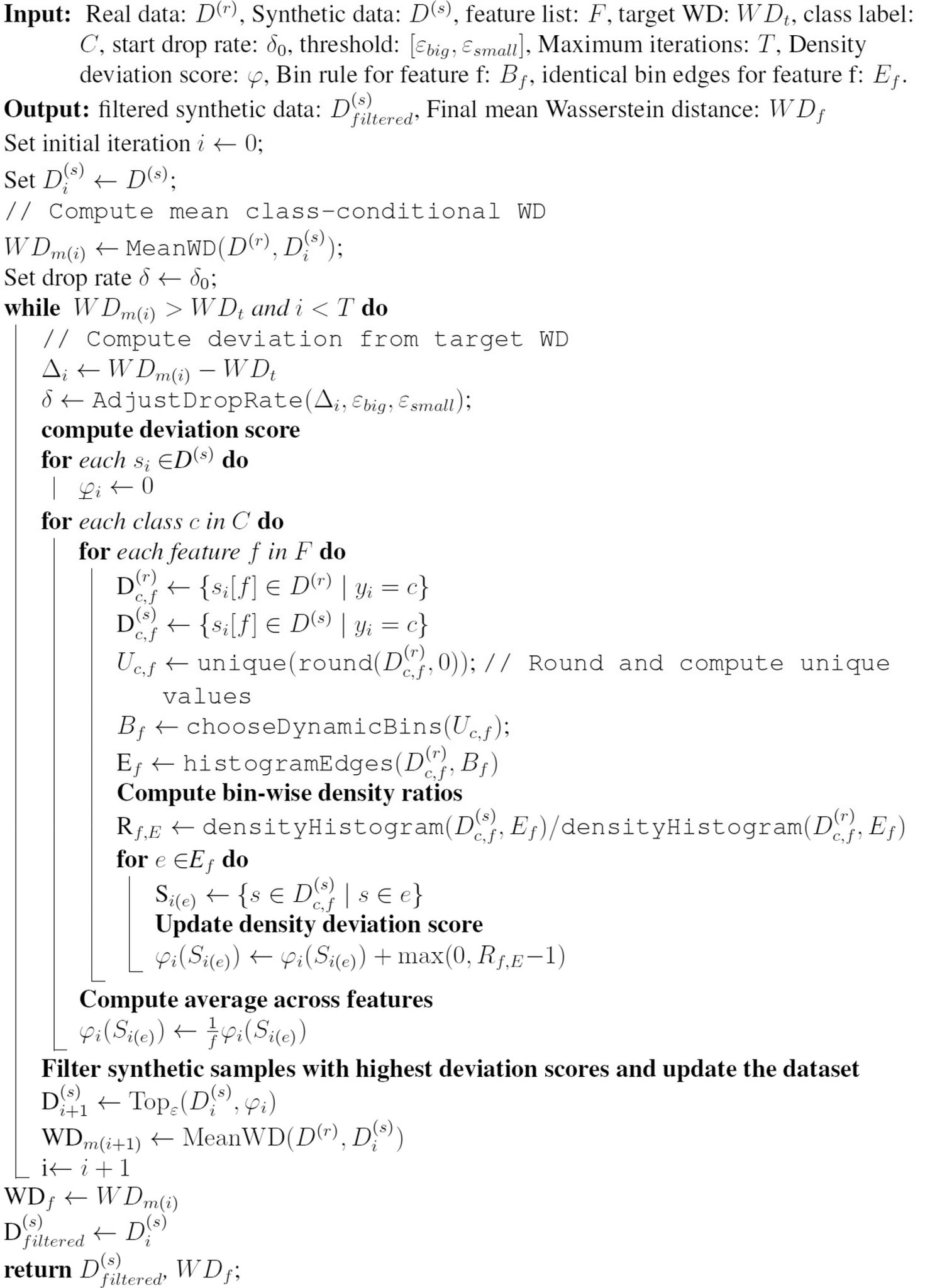

To facilitate reproducibility, we report the parameter values used in the experiments. This configuration was fixed across all runs. The discussed dynamic binning technique selected the following numbers of bins for the numerical features: Age (36), Albumin (6), Micro Cultures Count (3), Imaging Total Count (5), whereas “scott” rule was found as the optimal value for the Bicarbonate, Chloride, and Glucose features. For the adaptive filtering procedure, the start drop rate $\left(\delta_{0}\right)$ was set to 0.20, so the filter starts by dropping an acceptable range of data, and this value will be adjusted later, as illustrated in the algorithm. The lower and upper distance thresholds (ε) for adjusting the drop rate were set to 0.20 and 0.50, respectively. In addition, the maximum number of iterations was set to 30. So, the filtering procedure stopped when the fidelity criterion was satisfied by reaching the target WD, or the maximum iteration limit was reached. Besides, to prevent excessive data removal, an extra stop criterion was added to stop if the number of remaining synthetic records fell below the data size threshold (50% of the original synthetic dataset size).

Moreover, the final filtering step is model-based filtering. We simply pass the synthetic data to the trained model using the real data to filter out samples where the predicted class does not match the synthetic class. Then, we test combining different portions of real data with the full synthetic dataset to see which combination yields the best results.

### 3.6 Experimental setup

The whole experiment was implemented in Python, using various data science and machine learning libraries, including NumPy and pandas for data pre-processing, scikit-learn for model training and evaluation, and Matplotlib for visualization. Furthermore, the data has been split into a testing and a training set following an 80:20 ratio, resulting in 7,374 training samples and 1,846 testing samples. We employ stratified sampling, which is s a probability sampling method for dividing a population into distinct subgroups according to shared characteristics. This strategy preserves the original class distribution of the LOS outcome across both subsets. The resulting dataset was used for subsequent analyses, including prompt construction, baseline data generation, and the evaluation process.

#### 3.6.1 Baseline methods

Train on Real, Test on Real (TRTR) is the predictive model. It is trained and evaluated solely on real data and was adopted as an upper-bound estimate of the achievable utility. Furthermore, we utilize CTGAN and TVAE as baseline synthetic data generation models. CTGAN is a GAN-based approach, whereas TVAE is a Variational Autoencoder (VAE)-based tabular data synthesizer approach. Both models were trained using the same real training dataset to ensure a fair comparison with the proposed LLM-based approach. To ensure an unbiased comparison, both baseline models were trained using the same experimental settings and hyperparameter configurations, including 300 epochs and a batch size of 500. They generated the same number of records as the training set (7,374 records). The generated data were subsequently used for evaluation and comparison with LLM-based data. Additionally, for privacy preservation, the baseline model that achieved the strongest Pairwise Correlation Difference (PCD) performance is used to select few-shot samples for LLM guidance and to prepare training data for the fine-tuning process. The objective is to select samples that provide mostly realistic feature relationships and accurately preserve the correlation structure of the real dataset.

#### 3.6.2 Fine-tuning configuration

For the fine-tuning process, additional steps were required, including preparing the model training dataset and selecting the appropriate base model. The dataset consists of 1,000 samples extracted from the CTGAN-based generated data using NearestNeighbors, as we did for the few-shot example selection. Then we convert the dataset from a record format to a structured, text-based format suitable for fine-tuning. Since we are preparing data for the ChatGPT model, we followed the official ChatGPT fine-tuning format, using a message structure with system, user, and assistant roles. Accordingly, we prepared JSONL (JSON Lines) format, where each line is an independent training example involving the prediction class, since the fine-tuning process was designed to support both synthetic data generation and downstream prediction tasks. The fine-tuning process utilized the base model gpt-4.1-mini-2025-04-14, with a batch size of 5, a learning rate multiplier of 2, and 3 training epochs. These settings were selected based on preliminary experiments and the default recommendations provided by the platform.

### 3.7 Evaluation

We perform a fidelity evaluation to assess how closely synthetic data matches the real data in terms of statistical properties and patterns. Further, we use utility evaluation to measure how effectively the synthetic data can predict compared to real data.

For fidelity evaluation, we calculate the Wasserstein Distance (WD) to measure distribution similarities. It is also known as the Earth-Mover's distance. It is the cost of transforming the vector *u* into the vector *v*. The cost is calculated as the amount of probability mass being moved multiplied by the distance it is being moved (37). It is defined as:

$$l(u,v)=\int_{+\infty }^{-\infty }\left|U-V\right|$$

where *U* and *V* are the respective cumulative distribution functions of *u* and v

The small WD value indicates a good synthetic dataset that closely characterizes the real dataset. Since WD just works on numerical data, we compute the Jensen–Shannon (JS) Divergence for categorical features. JSD measures the similarity between two probability distributions as follows:

$$JSD(u,v)=\sqrt{\frac{D(\;p||m)+D(q||m)}{2}}$$

Where *m* is the pointwise mean of *u* and *v*, and D is the Kullback-Leibler divergence.

Both WD and JSD are univariate metrics. Besides, the density-based refinement works on improving the WD. Thus, we included additional metrics for fidelity evaluation.

We compute Discriminator-AUC for discriminator-based evaluation. It is an effective multivariate fidelity measure (48). Values close to 0.50 refer to high fidelity, where the synthetic data successfully mimics the real data distribution.

For privacy, we calculated two of the popular privacy metrics: Distance to Closest Record (DCR) and Nearest Neighbor Distance Ratio (NNDR) (49). DCR measures the Euclidean distance between each record in the synthetic and the nearest real data record. High values show greater separation between synthetic and real samples, while low values suggest a high risk of memorization. It is computed as:

$$\mathrm{DCR}(S,R)=\frac{1}{|S|}\underset{s_{i}\in S}{\sum }\;\underset{r_{j}\in R}{\min}\;\mathrm{dist}(s_{i},r_{j})$$

where *R* denotes the real dataset while *S* denotes the synthetic dataset, $\mathrm{dist}(s_{i},r_{j})$ is the Euclidean distance between a synthetic record $s_{i}$ and a synthetic record $r_{j}$, ∣S∣ is the number of synthetic records.

On the other side, NNDR computes the ratio between the synthetic record and the distance to its nearest neighbor and the distance to its second-nearest neighbor in real data. As in DCR, higher values generally indicate higher privacy. It is computed as:

$$\mathrm{NNDR}(s)=\frac{dist(s,r_{1})}{dist(s,r_{2})}$$

For utility evaluation, we apply Train-On-Synthetic, Test-On-Real. This method involves training a prediction model on synthetic data and testing it on real data. We trained a CatBoost model (50). It is based on decision trees. We chose this algorithm for its effectiveness in handling tabular data. It is a strong competitor to LightGBM and XGBoost. Unlike these algorithms, CatBoost does not require encoding since it can handle categorical data. Besides, it has demonstrated its capability for LOS prediction in previous work (19)**.** The CatBoost classifier was configured with 1,000 boosting iterations, a learning rate of 0.05, a tree depth of 6, a random seed of 42, and early stopping after 50 rounds without improvement. Subsequently, we evaluate the model using four multivariate utility measures: Macro-F1, Matthews correlation coefficient, Area Under the Precision–Recall Curve, and Receiver Operating Characteristic—Area Under the Curve (ROC-AUC).

Unlike Discriminator-AUC that used to distinguish the synthetic data from the real data, ROC_AUC measures how useful the synthetic data is for training downstream models, so the model is trained on synthetic and tested on real. Plotting the ROC curve requires calculating the True Positive Rate (TPR) vs. the False Positive Rate (FPR). TPR, also called a recall, is defined as:

$$TPR=\frac{True\;positives}{True\;positives+False\;negatives}$$

FPR is defined as:

$$FPR=\frac{False\;positives}{False\;positives+True\;negatives}$$

where the ROC-AUC is the area under this curve, it reflects the probability that a randomly chosen positive is graded with a higher score than a randomly chosen negative.

Subsequently, we use PR-AUC (Area Under the Precision–Recall Curve), which is a single number that summarizes the information in the PR curve. It is computed as:

$$\mathrm{PR}\text{-}\mathrm{AUC}=\overset{n-1}{\underset{i=1}{\sum }}\;(R_{i+1}-R_{i})P_{i+1}$$

where $R_{i+1}$ and $P_{i+1}$ are the recall and precision at the i^th^ threshold.

The other multivariate metric is Macro-F1. We select it because it is suitable for datasets with class imbalance, where it assigns equal weight to each class regardless of class frequency. It is calculated using the following formula:

$$\mathrm{Macro}\text{-}F1=\frac{1}{n}\overset{C}{\underset{x=1}{\sum }}\;f1_{x}$$

where *n* represents the number of classes, the F1-score of class *x* is calculated as:

$$f1=2\;\frac{Precision.\;Recall}{Precision+Recall}$$

Where recall is calculated using the TPR equation explained above, while precision is defined as:

$$precision\;=\frac{True\;positives}{True\;positives+False\;positives}$$

The Matthews correlation coefficient (MCC) is suitable for evaluating the performance of a binary classification model, especially when the class distribution is imbalanced. It is calculated using the following equation:

$$\mathrm{MCC}=\frac{TP\times TN-FP\times FN}{\sqrt{\left(TP+FP)(TP+FN)(TN+FP)(TN+FN\right)}}$$

where: TP (True Positives), TN (True Negatives), FP (False Positives), and FN (False Negatives).

Moreover, we calculate the pairwise correlation difference (PCD). It is a bivariate utility measure (51). It provides pairwise relationships among individual variables, yielding valuable insights. Additionally, it represents the difference between the correlations of the real and synthetic data. The average PCD for a set of features $F=\{\;f_{1},f_{2},\ldots ,f_{i}\}$ defined as:

$$Avg\_PCD(x_{real},x_{synth})=\frac{1}{i}\underset{\;f\in F}{\sum }\;|corr(x_{real}\;)-corr(x_{synth})|$$

Moreover, we compare histogram-based distributions by plotting histograms to provide a visual assessment of how closely the synthetic samples match the original feature distributions.

## 4 Results

The reported results for LLMs in all figures in this section, unless otherwise stated, represent the mean value across multiple independent generation runs. Error bars represent the 95% confidence interval (CI) calculated from the repeated experiments. The goal of adopting this evaluation strategy is to address the variability introduced by stochastic synthetic data generation. Mean and CI provide a more robust assessment of model performance.

The 95% CI was calculated using the following equation:

$$CI=\bar{x}\pm t_{value}\left(\frac{s}{\sqrt{n}}\right)$$

where $\bar{x}$ represents the mean across runs, *s* represents the sample standard deviation, and *n* is the number of runs. The $t_{value}$ depends on the number of synthetic generation runs. Since the Claude model has only two generations due to the high generation cost, the $t_{value}$ equals to 12.706, leading to wider confidence intervals, addressing the increasing uncertainty related to assessing variability from only two observations. Besides, the $t_{value}$ equals to 4.303 for the models with three runs.

**Figure 3:**
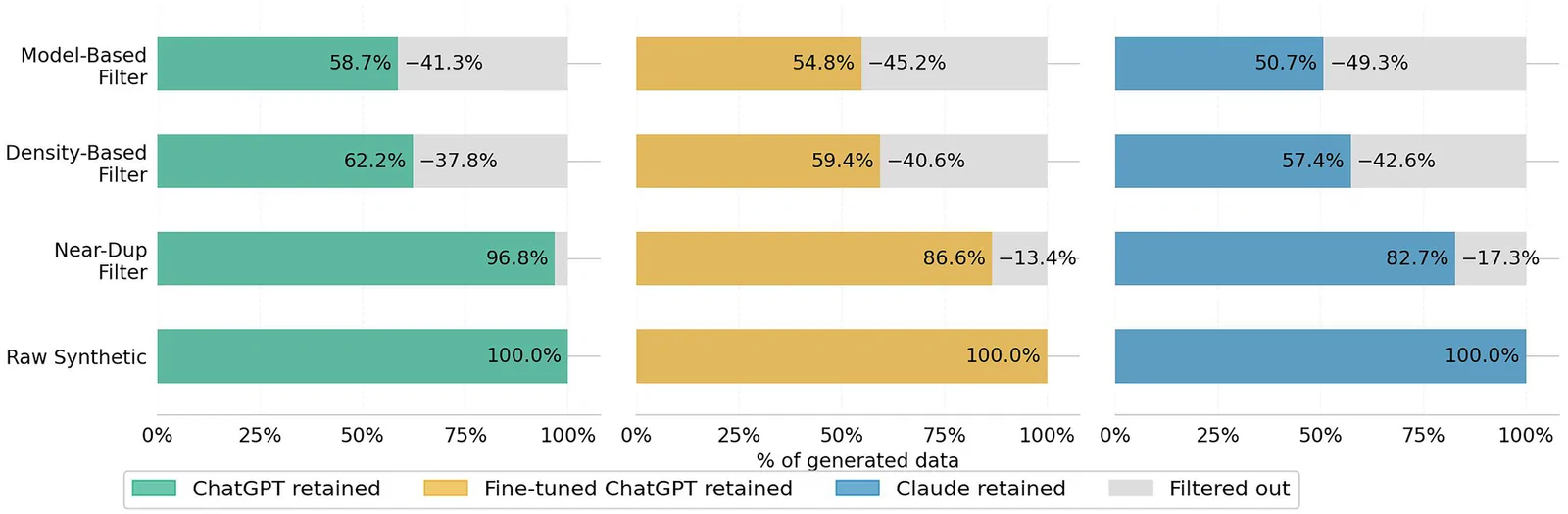
Changes in synthetic dataset size across post-processing filtering stages for LLM-based synthetic data generation.

Figure 3 illustrates the reduction in data size by each stage of the proposed post-processing pipeline across all three LLM-based models. Since we have multiple generation runs, we calculate the mean value per stage. All models begin at 100% of their generated output, considering they all generated the same amount of data, 4,000 records. Near-duplicate filtering eliminates a small proportion of redundant records, particularly for ChatGPT, with retention rates of 3.2%. In the next filtering stage, a substantial proportion of records are removed at a rate ranging from 37.8% to 42.6% across models, as this filtering stage discards samples whose feature distributions significantly differ from the real data distribution. The model-based filtering removed an additional 3.5% of records for ChatGPT, 4.6% for Fine-tuned ChatGPT, and 6.7% for Claude. All models show comparable overall reduction rates, with final reductions ranging from 41.3% for ChatGPT to 49.3% for Claude. This indicates that the suggested filtering pipeline applied a similar level of quality control across the models.

### 4.1 Fidelity and privacy evaluation

**Figure 4:**
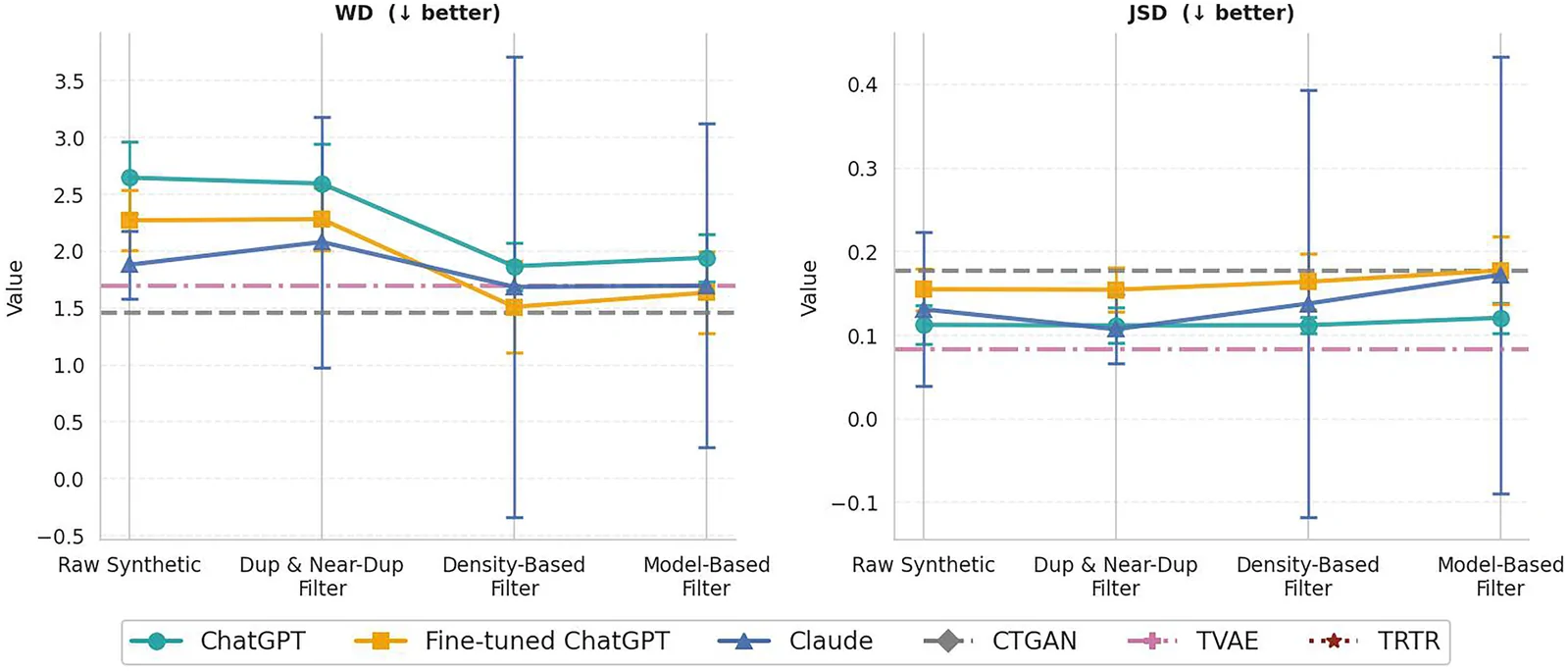
The univariate fidelity metrics: the average wasserstein distance and the average jensen–Shannon (JS) divergence.

Figure 4 illustrates the univariate fidelity evaluation results. It shows the average WD and average JSD for the selected LLMs across stages. The lower the value in these metrics, the better. Post-processing helps improve the average WD for numerical features. The density-based refinement step results in a major drop, while the model-based filter preserves the results or causes only a minor increase. However, the slight upward shift by the last post-processing step (model-based filter) is lower than the WD for raw synthetic data. The last post-processing step leads to a decrease in the average WD by ≈ 0.75% for ChatGPT and fine-tuned ChatGPT, and 0.18% for Claude. Subsequently, TVAE obtains an average WD of 1.7, whereas CTGAN obtains an average WD of 1.46, which is similar to the value achieved by Fine-tuned ChatGPT after Density-based refinement.

Moreover, the average JSD is used for the categorical features (First Care-Unit and Admission Departments). The effect of the post-processing steps varies depending on the model. The filtering leads to a significant increase in JSD for the Claude model, and a slight upward shift for Fine-tuned ChatGPT, particularly after the model-based filter, reflecting reduced alignment. Although post-processing does not improve JSD values, these values represent only two features. Subsequently, the benefit of these filtering steps is significant for the remaining numerical features. Furthermore, CTGAN obtained a JSD of 0.17, which is worse than ChatGPT and Fine-tuned ChatGPT in most phases. On the other side, TVAE obtained JSD of 0.08, which is the best value.

**Figure 5:**
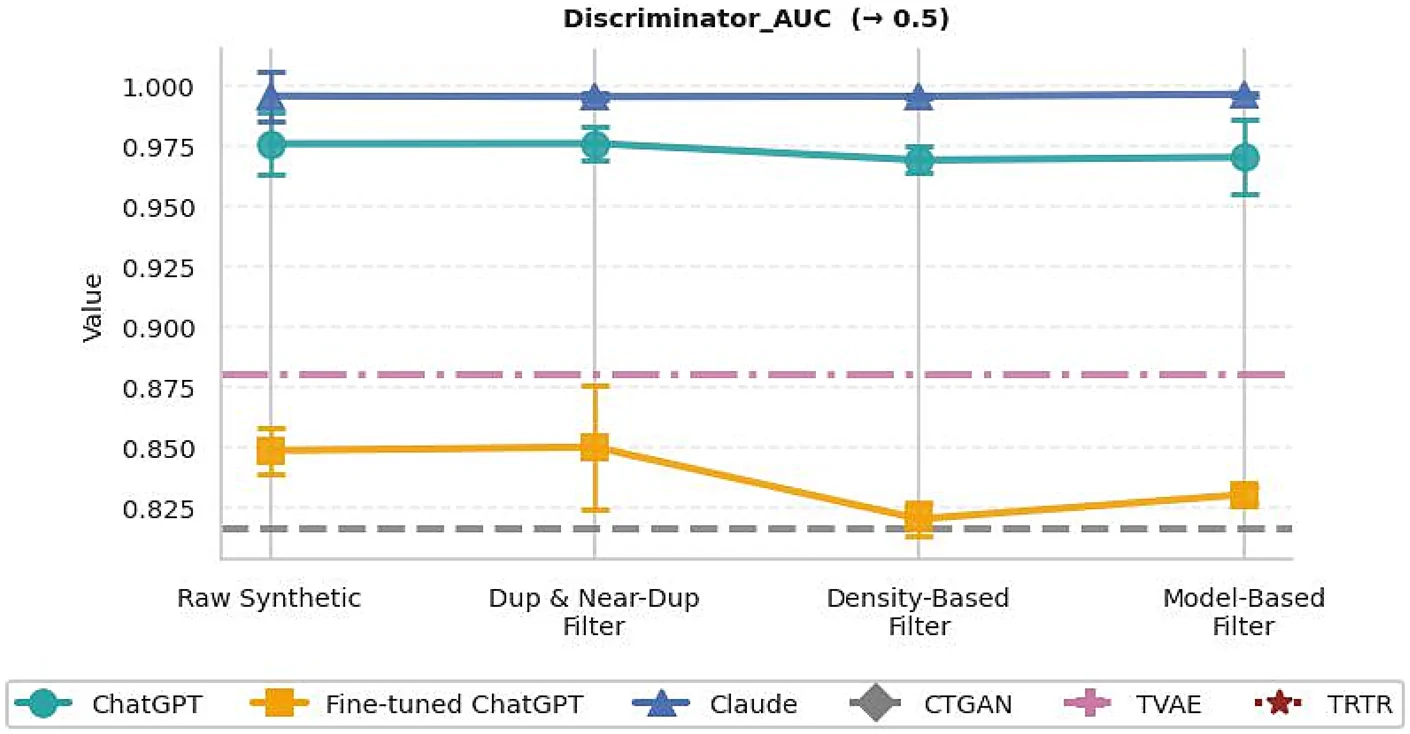
The multivariate fidelity metrics: discriminator-AUC.

Figure 5 shows Discriminator-AUC for Discriminator-based evaluation. Fine-tuned ChatGPT obtained the lowest value among the LLM models, indicating the best value. However, although CTGAN achieved the lowest values, Fine-tuned ChatGPT reached a comparable Discriminator-AUC value at Stage 3. However, following the transition to Stage 4 (Model-based Filtering), Discriminator-AUC increased. This suggests that the final filtering stage may have altered the data distribution, resulting in a slight degradation in this metric despite improvements in other evaluation measures. Similar to WD Metric, the proposed Density-based filtering strategy resulted in a slight improvement compared with the raw synthetic.

**Figure 6:**
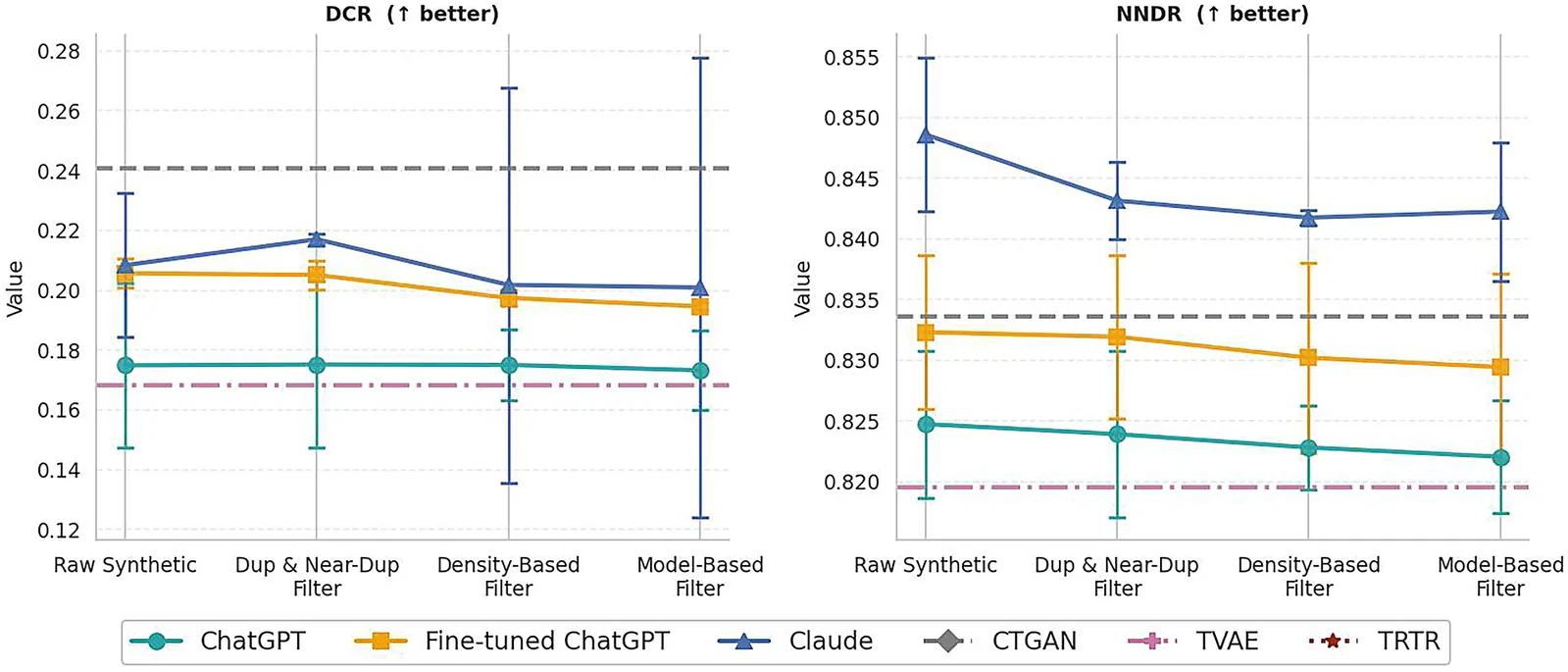
Privacy evaluation using distance to closest record (DCR) and nearest neighbor distance ratio (NNDR).

Figure 6 illustrates the privacy evaluation using DCR and NNDR across the different generation stages and baseline models. Higher values generally indicate greater separation from real data. The privacy evaluation results show that the Claude model consistently achieved the best results across both DCR and NNDR compared with the other LLMs. While the raw synthetic data obtained the highest NNDR values, a slight reduction was observed after the final filtering stage, indicating that the post-processing steps improved data quality at the cost of some separation from real records. Nevertheless, the final-stage results by Claude outperform the baseline methods in NNDR. However, CTGAN achieved the highest DCR scores. It is important to mention that the LLMs were not provided with direct real patient records during generation. Even in the few-shot setting, the examples passed to the models were synthetic records generated by baseline methods instead of actual patient data. Notably, the changes observed across post-processing stages are more likely attributable to the filtering and refinement procedures than to memorization of real records.

### 4.2 Utility evaluation

**Figure 7:**
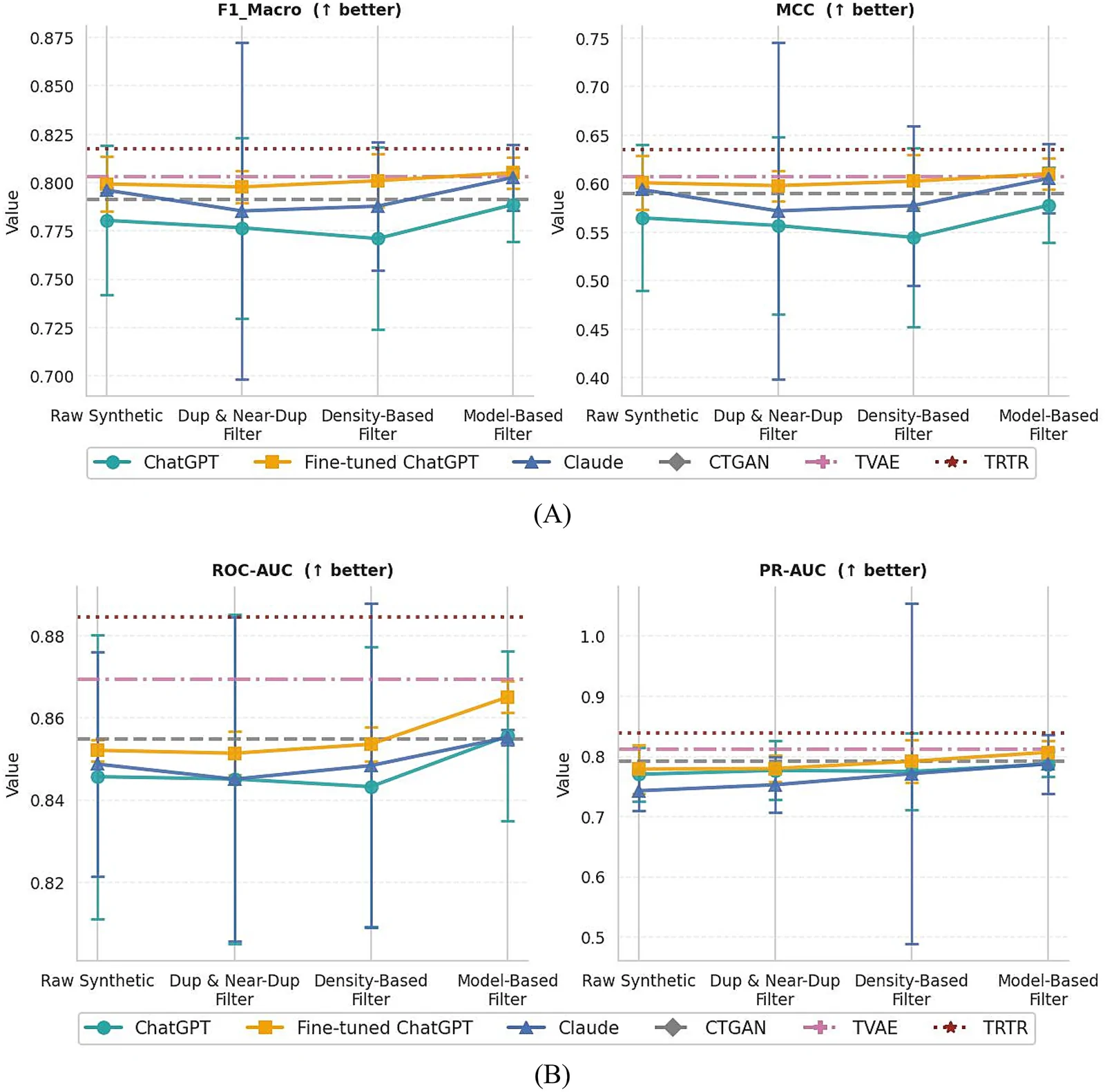
Comparative utility evaluation of synthetic datasets across different LLMs. **(A)** Label-Based Metrics: Macro-F1, MCC. **(B)** Probability-Based Metrics: ROC-AUC, PR-AUC.

Moreover, Figure 7 shows utility evaluation results for Train on Real Test on Real (TRTR) and synthetic data across different LLMs compared with the baseline (CTGAN and TVAE models). The figure illustrates the results across the post-processing stages to explore how these steps could improve performance. Overall, the utility performance remained stable throughout the stages, with gradual improvements observed for most models, while a slight decrease by some models was noted following the near-duplicate filtering stage, or the Density-based filtering. The reduction was minimal and was subsequently recovered in the later refinement stages.

The final-stage results demonstrate that the proposed refinement strategy improves data fidelity without negatively affecting predictive utility. Among the evaluated models, Fine-tuned ChatGPT and Claude achieved the strongest performance, producing results comparable to TVAE across Metrics: F1-Macro, MCC, and PR-AUC. The only noticeable difference was observed in PR-AUC, where Claude scored 0.01 lower than them. Although TVAE achieved the highest value for ROC-AUC, outperforming Fine-tuned ChatGPT by 0.04, the difference was relatively small compared with the overall similarity observed across the other utility metrics.

**Figure 8:**
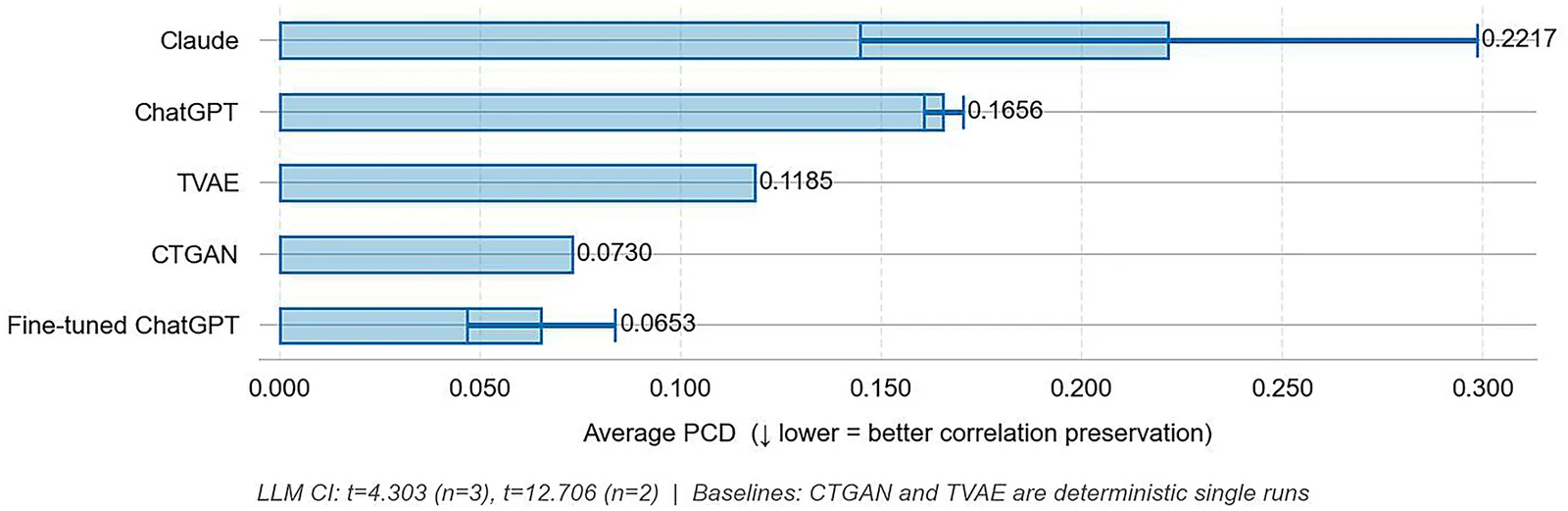
Average pairwise correlation difference (PCD).

Overall, the utility performance of the LLM-based models slightly improved throughout the refinement stages. Despite ChatGPT showing slight decreases after certain filtering steps, these reductions recovered in later stages. Notably, the utility performance of the final stage (Model-based Filtering) exceeded that of the raw synthetic data, indicating that the proposed refinement process did not adversely affect downstream predictive performance. Furthermore, Figure 8 illustrates the average PCD. A lower PCD value is better, indicating that the synthetic data more closely aligns with the real data in terms of feature–label dependency strength. Among the evaluated methods, Fine-tuned ChatGPT produced the lowest PCD score of 0.065, suggesting superior preservation of the underlying correlation structure.

**Figure 9:**
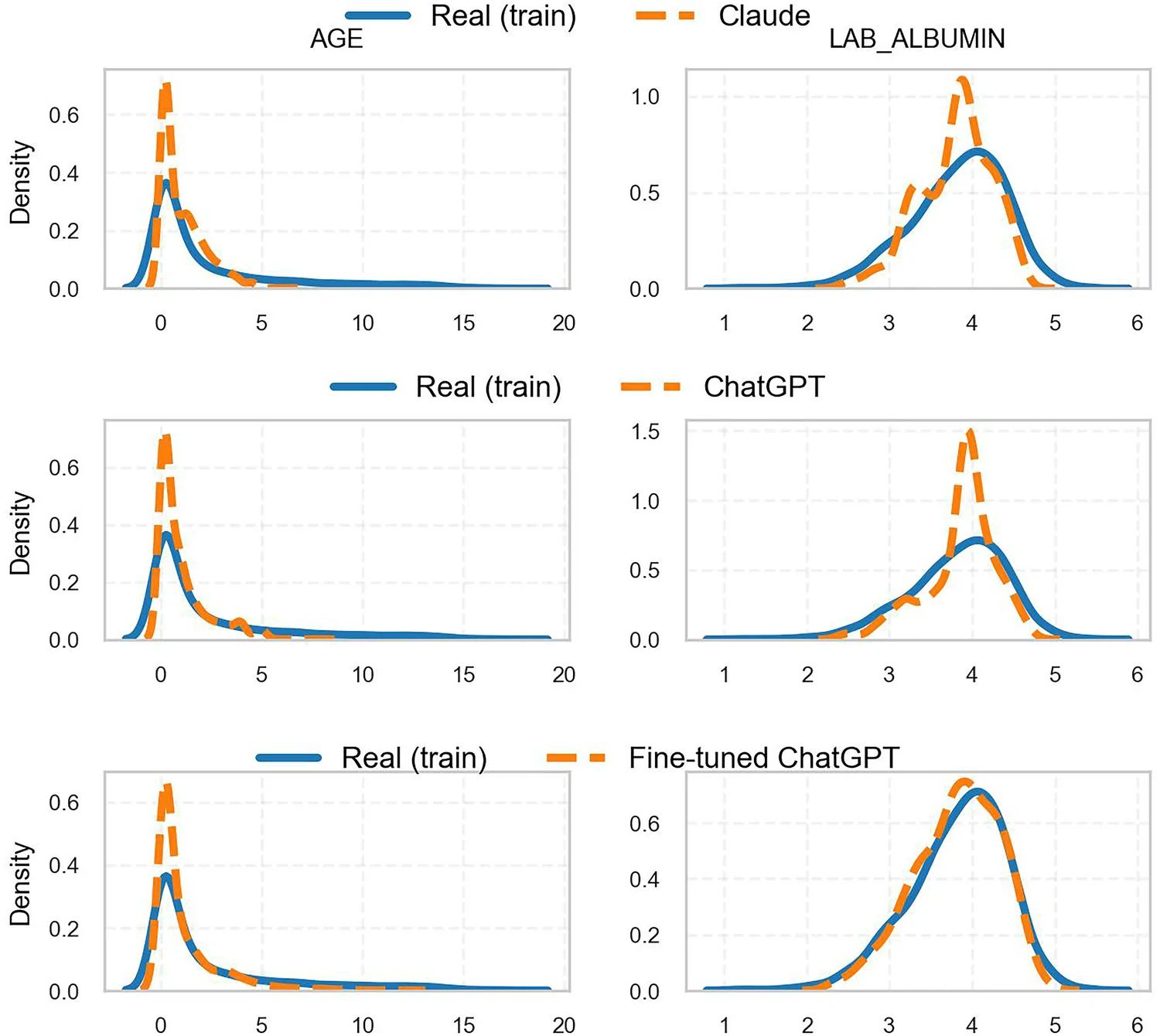
Comparison of selected continuous features distributions between real and LLM-based synthetic data.

**Figure 10:**
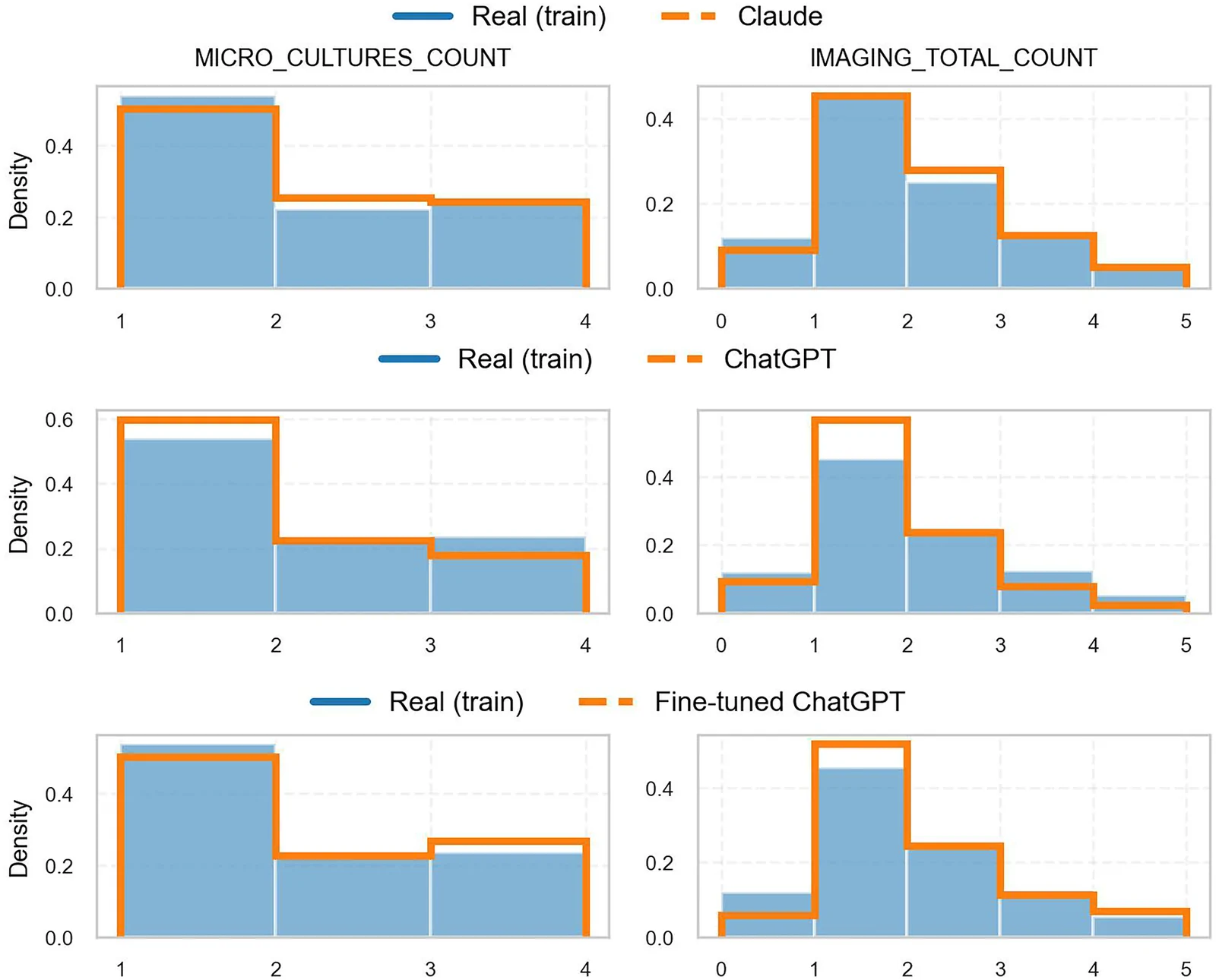
Comparison of selected discrete feature distributions between real and LLM-based synthetic data.

To ensure that synthetic data preserves feature-level statistics. The plots in Figures 9, 10 illustrate the numerical distributions of real and synthetic data. We included the final data we got after post-processing. To enhance clarity and interpretability, rather than plotting all features for all selected models, we visualize only the selected features. We pick the most influential features as ranked by the Random Forest classifier. For each LLM, we select the run whose values were closest to the mean across all runs. It can be noticed that the synthetic data generated by the Fine-tuned ChatGPT model more closely follows the real data distribution in terms of overall shape and fairly preserves the features' skewness compared to the other models. On the other hand, Claude does not perfectly match the shape and skewness, even though it also obtained high scores in the utility evaluation metrics, indicating that the distribution shape does not necessarily reflect the model's predictive success.

**Figure 11:**
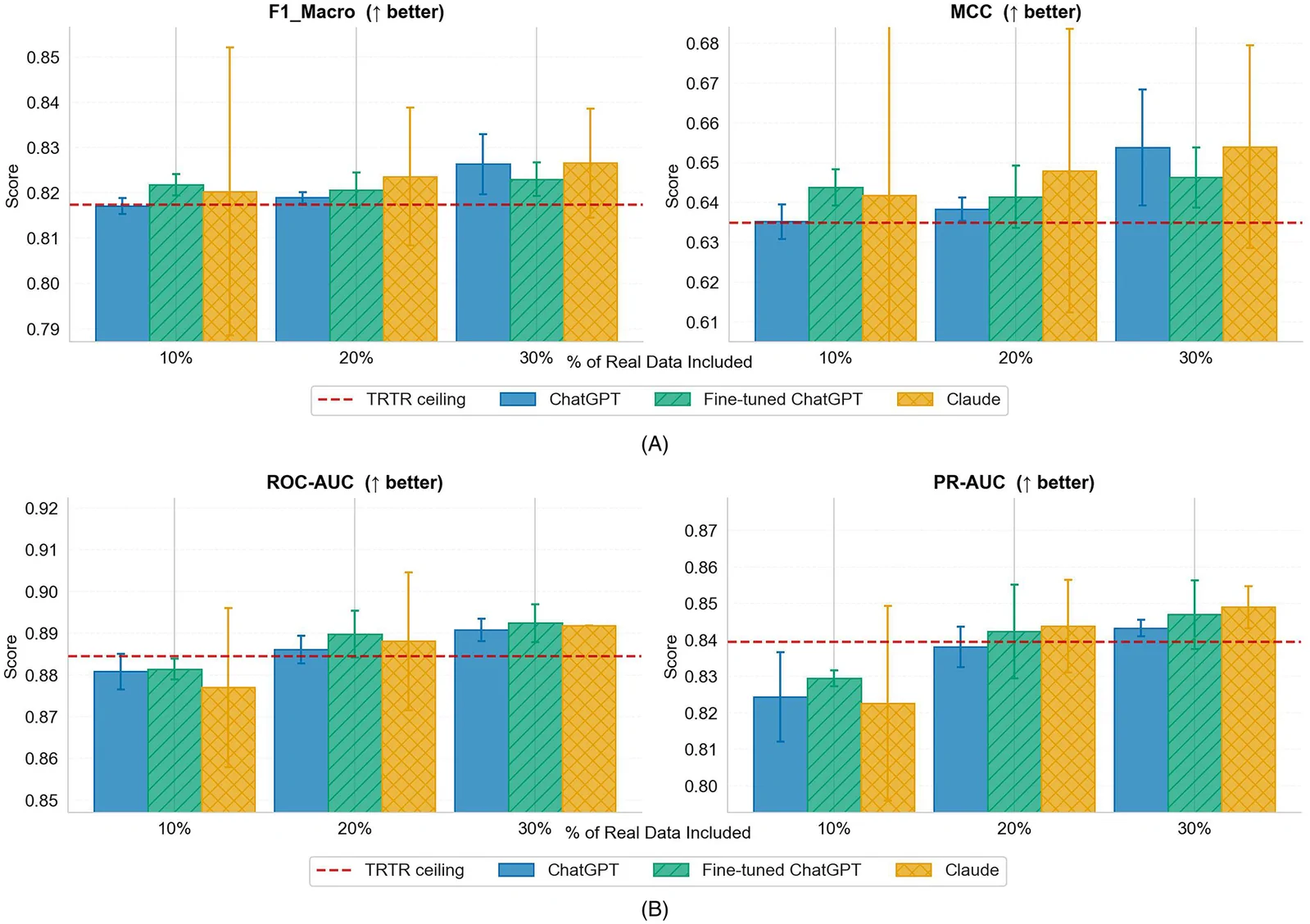
Effect of integrating real data with filtered synthetic data on classification utility. **(A)** label-based metrics: Macro-F1, MCC. **(B)** Probability-based metrics: ROC-AUC, PR-AUC.

Figure 11 presents the effect of merging the whole synthetic data after the filtering process, with increasing proportions of real data across all four utility metrics. The objective of the merged-data experiments was to investigate whether generated synthetic data could effectively complement a small ratio of real data, reflecting a real constraint due to clinical data scarcity and privacy concerns. We employ stratified sampling to ensure an equal proportion for both classes. Since we have multiple synthetic run generations, we tested integration with each synthetic run, and then the mean value is calculated. The red line represents the pure Train-on-Real/Test-on-Real (TRTR) evaluation, considering training on full real training data. As the ratio of real data increases, all the models show improvement across metrics, exceeding the real-data baseline. Notably, starting from 20% real data, all models surpass the performance of real data alone across almost all metrics. These results indicate that the proposed filtering pipeline led to the generation of synthetic data that meaningfully complements real data, handling the scarcity issue.

## 5 Discussion and limitations

We applied multiple evaluation measures to assess the quality of the synthetic data generated. Univariate measures, such as WD and JSD, assess the similarity between synthetic and real data. However, they focus on a single feature and cannot capture the complex structure dependencies between features. In contrast, multivariate measures such as Discriminator_AUC assess whether joint feature structure is preserved. Besides, bivariate measures such as pairwise correlation difference (PCD) assess the relationship between pairs of features and indicate whether synthesis can preserve these relationships. On the other hand, multivariate measures such as ROC-AUC, PR-AUC, and Macro-F1 can evaluate the joint distribution of three or more features. Therefore, they enable deeper insight into both patterns and structures.

Claude achieved the best DRC and NNDR among the other LLMs. However, a modest reduction in DCR and NNDR was observed during the filtering stages. This behavior is expected because the refinement process discards records that are less representative of the underlying real-data distribution, leading to improved fidelity. Despite this reduction, the resulting distances remained sufficiently large, suggesting that the synthetic records were not direct replicas of real patients. Most importantly, none of the LLMs were exposed to real patient examples during generation or fine-tuning, where the few-shot examples were derived from baseline-generated synthetic data rather than real records.

Moreover, by examining the results from the final post-processing step (model filtering), we find that Fine-tuned ChatGPT outperforms the other LLMs in terms of Discriminator_AUC, while demonstrating competitive performance with Claude on the WD metric. Subsequently, the utility performance of Fine-tuned ChatGPT and Claude was largely similar, with both models achieving equivalent results across Macro-F1, PR-AUC, and MCC. Nevertheless, Fine-tuned ChatGPT maintained a slight advantage by achieving a superior result on ROC-AUC and average PCD.

Notably, despite the smaller number of synthetic records, particularly after refinement, Fine-tuned ChatGPT and Claude achieved utility performance comparable to that of the baseline method across Macro-F1, MCC, and PR-AUC. Besides, Claude outperforms the baseline in the NNDR measure, while Fine-tuned ChatGPT outperforms them in the PCD metric. This suggests that the quality of the generated samples may be more important than the quantity alone. Subsequently, the results of the visual density are not equal to quantitative fidelity; thus, the model may look visually aligned but score worse quantitatively. This explains why Claude does not fully preserve the shape of all features as the Fine-tuned ChatGPT model does, but it surpasses it in the utility and fidelity evaluation metrics.

As expected, none of the synthetic-data approaches surpass the utility results obtained from the model trained on real data, which serves as the reference upper bound. Since synthetic datasets are generated using data derived from the original dataset, achieving utility metrics that closely resemble those of real data can be considered as evidence of effective distribution preservation during the generation process. Subsequently, the finding indicates that integrating a small ratio of real samples with filtered synthetic data can outperform real data alone. This emphasizes that the generated data not only replicate real samples but also lead to generating valuable samples, and thus combining them with real samples leads to improved utility results.

In summary, the results demonstrate that integrating prompt-guided statistical modeling with the proposed post-processing pipeline, particularly the density-aware refinement, improves the alignment between generated and real data. An empirical evaluation of pediatric ICU length-of-stay datasets confirms that the proposed framework achieved improved statistical alignment and competitive predictive performance. These findings highlight the potential of the proposed framework in addressing data scarcity in critical domains.

Despite these promising findings, several limitations should be acknowledged. The proposed framework was evaluated on specific pediatric EHR datasets, which may limit external validity. The study focuses primarily on structured data and does not evaluate the incorporation of unstructured clinical text. Additionally, the work is limited to the selected LLMs, and results may vary with different large models or newer versions. Additionally, generating large-scale synthetic data may lead to an increase in financial costs. Moreover, the proposed framework's design is generalizable, ensuring applicability across diverse prediction tasks and clinical conditions. However, the framework depends on predefined rules and constraints, which may require involving domain experts to refine constraints and validate the removal of clinically incorrect or out-of-range data. Future work is required to mitigate the limitations and confirm robustness. Furthermore, in this study, the missing values were removed during preprocessing. In real clinical datasets, missingness, particularly for lab tests, provides meaningful data regarding patient condition. Future work should consider methods for generating missingness patterns in synthetic data. Another limitation of this work is fairness or subgroup-specific performance evaluation. Thus, future work should examine whether the generated synthetic data preserves data characteristics across different patient subgroups. Additionally, although mean performance and 95% confidence intervals are reported to handle stochastic variation from LLM sampling, the number of generations run per model conducted in this paper is insufficient to support statistical significance testing. Therefore, future studies should comprise a larger number of independent runs to support hypothesis testing.

## 6 Conclusion and future work

This work proposes an LLM-based synthetic data generation framework integrating a rule-guided prompting method and advanced post-processing strategies. We adopt a class-aware rule generation method to ensure that the generated data aligns with the class-specific distributional patterns and preserves class–feature relationships. We integrated feature importance analysis and the feature-feature interaction method. To avoid distracting the prompt with multiple rules, we applied a ranking method to identify the high-influence features. Besides, we integrated empirical statistics, including mean, median, range bounds, and quantile thresholds, to characterize marginal distributions. Subsequently, to preserve structural dependencies, we use pairwise feature relationships based on correlation coefficients and mutual information scores, which enable the model to learn both linear and nonlinear interactions. Furthermore, we improve the quality of the generated data by applying multi-step post-processing, including near-duplicate filtering based on the SimHash function to increase diversity, density-aware refinement to drop unrealistic samples and enhance fidelity, and model-based filtering to discard synthetic samples inconsistent with real patterns.

Moreover, the findings demonstrate the effectiveness of the proposed pipeline in improving alignment between synthetic and real data. Besides, incorporating prompt-guided statistical rules effectively guides LLMs and thus supports their suitability for both generation and prediction tasks. Fine-tuned ChatGPT achieved the best performance in terms of average PCD, whereas Claude maintained an advantage in the NNDR metrics. Furthermore, both models demonstrated results comparable to baselines (CTGAN and TVAE) in terms of Macro-F1, PR-AUC, and MCC, despite retaining significantly fewer records following the refinement stages. This indicates that the proposed post-processing strategy effectively removed lower-quality samples while preserving informative records, resulting in a more efficient synthetic dataset. Overall, these results highlight the success of the proposed method in addressing the data scarcity and generating reliable synthetic data in such risk-sensitive domains.

Future work will extend the framework to multimodal data, including unstructured clinical notes and imaging. Subsequently, we will explore the generalization of the proposed framework across diverse domains. Additionally, the current prompt design is structured but manually engineered. Thus, automated prompt-optimization strategies could be explored to dynamically adjust statistical rule injection. An interesting direction for future research is introducing an approach to efficiently generating multi-valued clinical features, such as imaging examinations, rather than being aggregated into summary features. Furthermore, evaluating the framework on additional real clinical datasets represents an important direction for further investigation. Including computational cost and runtime analyses, along with evaluating data quality and predictive utility, will provide a more comprehensive assessment of the efficiency of diverse synthetic data generation methods.

## Data Availability

Publicly available datasets were analyzed in this study. This data can be found here: https://physionet.org/content/picdb/1.1.0/.
