# Supplementary material for: Adaptive refinement and prompt-guided conditioning for clinically realistic LLM-generated synthetic pediatric data
Source: Front Digit Health. 2026 Aug 19;8:1826526. doi: 10.3389/fdgth.2026.1826526 (PMC13533997; doi:10.3389/fdgth.2026.1826526)
Supplement: Supplementary file 1 [file Datasheet1.pdf]

Supplementary Material S1

Representative Prompt Template

Instruction

Model role

Produce exactly {n_records} JSON records, representing synthetic PICU stays.

Output structure

-**Output strict JSON using only double quotes for all keys and string values**.

-** Do not use single quotes or Python-style dicts**.

-** Do not include any text, explanations, or white space outside the JSON array**.

Feature definition

-**Each record must conform to this schema**:

'AGE', 'MICRO_CULTURES_COUNT', 'IMAGING_TOTAL_COUNT', 'lab_Albumin', 'lab_Bicarbonate', 'lab_Chloride', 'lab_Glucose','FIRST_CAREUNIT', 'ADMISSION_DEPARTMENT', 'LOS_category'

Note: LOS_category": must be 0 or 1, where 0=short (≤3d), 1=long (>3d).

Categorical feature

-**For categorical fields ("FIRST_CAREUNIT","ADMISSION_DEPARTMENT")**:

- "FIRST_CAREUNIT": only take one of the following: ['SICU', 'PICU', 'General ICU', 'NICU', 'CICU']

...

Mandatory constraints

- Use the exact spelling and capitalization as given. Do not invent new categories, abbreviations, or alternative spellings.

- Do not leave these fields empty.

- Do not generate any values outside the given options.

-** Ensure each record's "LOS_category" is backed by its features—apply rules/guides, so LOS=0 records reflect short-stay patterns and LOS=1 reflect long-stay patterns**

Class-specific rules and constraints

** LOS_category = 0 **

High-impact features

-Sample "MICRO_CULTURES_COUNT" from its distribution: 1 (66%), 2 (19%), 3 (14%)

...

Feature–Feature Interaction Rules

- pick a (FIRST_CAREUNIT → ADMISSION_DEPARTMENT) pair **sampled** from this top distribution:

  • CICU → Cardiothoracic Surgery 15.4%

  ...

- Sample "age" within the given statistical ranges for "ADMISSION_DEPARTMENT", where Q1 is the 25th percentile and Q3 is the 75th percentile:

  • Cardiothoracic Surgery: Q1=0.8, median=1.8, Q3=4.1

  ...

Correlation rules

•"age" and "lab_Albumin" have a moderate positive correlation.

...

Domain-specific information

AGE: 73% [0–3.6], 14% [3.6–7.1], 7% [7.1–10.7], 5% [10.7–14.3], <1% >14.3

...

** LOS_category = 1 **

High-impact features

- Sample "MICRO_CULTURES_COUNT" from its distribution: 1 (36%), 2 (26%), 3 (37%)

...

Feature–Feature Interaction Rules

- pick a (FIRST_CAREUNIT → ADMISSION_DEPARTMENT) pair **sampled** from this top distribution:

  • NICU → ICU (14.3%)

  ...

- Sample "age" within the given statistical ranges for "ADMISSION_DEPARTMENT":

  •Cardiothoracic Surgery: Q1=0.2, median=0.4, Q3=0.9

  ...

Correlation rules

•"age" and "lab_Albumin" have a moderate positive correlation.

...

Domain-specific information

AGE: 84% [0–3.4], 7% [3.4–6.8], 5% [6.8–10.2], 4% [10.2–13.6], 1% >13.6

...

Illustrative examples

Examples for LOS classification:

1-{"FIRST_CAREUNIT": "SICU", "AGE":0.34," ADMISSION_DEPARTMENT":"General Surgery","MICRO_CULTURES_COUNT":1,"IMAGING_TOTAL_COUNT":1,"lab_Albumin":4.14," lab_Bicarbonate":20.31,"lab_Chloride":111.36,"lab_Glucose":128.37,"LOS_category":0}

...

6- {"FIRST_CAREUNIT": "CICU", "AGE":1.03," ADMISSION_DEPARTMENT":"Cardiothoracic Surgery","MICRO_CULTURES_COUNT":3,"IMAGING_TOTAL_COUNT":1,"lab_Albumin":3.55," lab_Bicarbonate":23.05,"lab_Chloride":105.67,"lab_Glucose": 145.46,,"LOS_category":1}

Note

This supplementary material provides a representative prompt template. The exact numerical values (e.g., quartiles, probability distributions, and correlations) are dataset-dependent and were automatically extracted from the training data. Therefore, placeholders are used in place of the complete prompts while preserving the overall prompting strategy.
